# Drivers of beta diversity in modern and ancient reef-associated soft-bottom environments

**DOI:** 10.7717/peerj.9139

**Published:** 2020-05-14

**Authors:** Vanessa Julie Roden, Martin Zuschin, Alexander Nützel, Imelda M. Hausmann, Wolfgang Kiessling

**Affiliations:** 1 GeoZentrum Nordbayern, Section Paleobiology, Friedrich-Alexander University Erlangen-Nürnberg, Erlangen, Germany; 2 Department of Palaeontology, University of Vienna, Vienna, Austria; 3 SNSB—Bayerische Staatssammlung für Paläontologie und Geologie, Munich, Germany; 4 Department of Earth & Environmental Sciences, Ludwig-Maximilians-Universität München, Munich, Germany; 5 GeoBio-Center, Ludwig-Maximilians-Universität München, Munich, Germany

**Keywords:** Beta diversity, Macrobenthos, Reef basins, Mollusks, Community assembly, Triassic, Red Sea

## Abstract

Beta diversity, the compositional variation among communities, is often associated with environmental gradients. Other drivers of beta diversity include stochastic processes, priority effects, predation, or competitive exclusion. Temporal turnover may also explain differences in faunal composition between fossil assemblages. To assess the drivers of beta diversity in reef-associated soft-bottom environments, we investigate community patterns in a Middle to Late Triassic reef basin assemblage from the Cassian Formation in the Dolomites, Northern Italy, and compare results with a Recent reef basin assemblage from the Northern Bay of Safaga, Red Sea, Egypt. We evaluate beta diversity with regard to age, water depth, and spatial distance, and compare the results with a null model to evaluate the stochasticity of these differences. Using pairwise proportional dissimilarity, we find very high beta diversity for the Cassian Formation (0.91 ± 0.02) and slightly lower beta diversity for the Bay of Safaga (0.89 ± 0.04). Null models show that stochasticity only plays a minor role in determining faunal differences. Spatial distance is also irrelevant. Contrary to expectations, there is no tendency of beta diversity to decrease with water depth. Although water depth has frequently been found to be a key factor in determining beta diversity, we find that it is not the major driver in these reef-associated soft-bottom environments. We postulate that priority effects and the biotic structuring of the sediment may be key determinants of beta diversity.

## Introduction

Beta diversity, the compositional variation among communities, is a key aspect of biodiversity and of great interest to community ecologists. In paleontology, differences in community composition are often recognized but not always quantified. There are only few studies that address beta diversity in fossil assemblages and allow for a comparison with modern data. Variations in taphonomy, sampling intensity, and fossil availability usually impede comparisons between fossil and modern assemblages (Koch & Sohl, 1983; Raup, 1972; Smith & McGowan, 2011). An intuitive solution is analyzing very well-preserved assemblages and comparing skeletal fossils with modern assemblages from a similar environmental setting.

Disentangling the drivers of beta diversity in various habitats has been the aim of many biodiversity studies, most often ascribing variation in community composition to environmental factors and/or spatial distance (e.g., Melo, Rangel & Diniz-Filho, 2009; Qian & Ricklefs, 2007; Svenning, Fløjgaard & Baselga, 2011; Pitacco et al., 2019). In metacommunity theory, both species-sorting as well as mass-effect paradigms assume environmental and spatial heterogeneity as drivers of community dissimilarity (Leibold et al., 2004). For example, in many cases, topographic complexity plays a key role in community dissimilarity (Al-Shami et al., 2013; Ellingsen & Gray, 2002). Particularly in reefs, which are known for their topographic complexity, environmental heterogeneity or gradients have been established as the most important drivers of beta diversity (Becking et al., 2006; Carlos-Júnior et al., 2019; Pandolfi, 1996; Pandolfi, 1999; Pandolfi & Jackson, 2006). Unfortunately, diversity in studies of coral reefs may not be directly comparable to other marine studies due to differences in taphonomy and sampling strategy (Pandolfi & Minchin, 1995).

There is also large variation in faunal composition in very uniform habitats, such as the continental shelf (e.g., Ellingsen, 2001). While it is clear that environmental factors are a strong driver of beta diversity, regional diversity in connection with stochastic processes (such as random dispersal) (Stegen et al., 2013), biotic interactions, such as predation (e.g., Huntley & Kowalewski, 2007; Klompmaker & Finnegan, 2018; Stanley, 2008), and intrinsic factors related to organism characteristics, such as body size or dispersal rate (Soininen, 2010), may also play an important role. Finally, large-scale patterns in modern settings can result from regional-historical processes (“priority effects”—the order in which taxa arrive, e.g., Lawler & Morin, 1993) as well as environmental gradients (Rex, Etter & Stuart, 1997). This complexity can obscure the identification of key determinants.

Variability in community composition also depends on spatial scale and resolution (e.g., Barton et al., 2013; Ellingsen, 2001; Mac Nally et al., 2004; Pandolfi, 2002), making it difficult to compare beta diversity between different studies and environments. Pandolfi’s (2002) three-phase model demonstrates high faunal variability in coral reefs at small spatial and temporal scales and relatively high variability on large scales, but lowest variability at intermediate scales. Using simple pairwise measures of dissimilarity decreases the influence of spatial scale and resolution (Marion, Fordyce & Fitzpatrick, 2017; Soininen, 2010). Gamma (regional) diversity—related to the size of the dataset and overall diversity in the region—and uneven sampling can influence measured beta diversity; comparing results with a null model can help assess this effect (e.g., Astorga et al., 2014; Kraft et al., 2011; Segre et al., 2014).

Here we address whether water depth is the main driver of beta diversity in soft-bottom reef-associated assemblages, or whether other factors are more likely to drive faunal heterogeneity. Several studies have found differences in beta diversity in soft-bottom mollusk assemblages with depth, but patterns are not uniform (Aldea, Olabarria & Troncoso, 2009; Benkendorfer & Soares-Gomes, 2009; Koulouri et al., 2006). Soft-bottom reef basin habitats are well-suited to explore this question due to their lack in complex topography—as opposed to the reefs themselves –and easier assessment of environmental variables, although there can be strong habitat variability in these environments, such as differences in sediment grain size and coverage by algae or seagrass. We investigate beta diversity patterns in a Triassic reef basin assemblage from the Cassian Formation in the Dolomites and compare results with a Recent reef basin assemblage from the Northern Bay of Safaga, Red Sea (Zuschin & Hohenegger, 1998; Zuschin & Oliver, 2005).

Determining the drivers of faunal heterogeneity has long been one of the central questions in ecology—how communities assemble in an ecosystem (Remmer et al., 2019; Stegen et al., 2013). However, more standardized and therefore comparable datasets are needed to help disentangle the drivers of beta diversity (Keil & Chase, 2019). With the ancient Cassian and the modern Safaga communities sharing several patterns of beta diversity, our results help to provide a better understanding of diversity patterns in warm-water reef-associated faunas. To discern the factors contributing to differences in community composition, we evaluate beta diversity with regard to known variables: geological age, water depth, and spatial distance. We then compare the results with a null model to evaluate the stochasticity of these differences.

## Materials & Methods

### Triassic data

The Cassian Formation in the Dolomites, Southern Alps, northern Italy, preserves Middle to Late Triassic (Ladinian–Carnian) tropical reef to basin environments with exceptional fossil preservation, allowing comparisons with recent assemblages due to a low taphonomic bias (Roden et al., 2020). The Cassian Formation comprises deposits with considerable differences in depth, from back-reef and lagoonal settings as well as shallow and deeper water deposits from the reef basin (e.g., Bosellini, 1998; Fürsich & Wendt, 1977). The platform-to-basin relief has been proposed to have exceeded 100 m, possibly even reaching bathyal depths (Keim et al., 2001; Urlichs, 2012). The predominantly argillaceous basin sediments were deposited between prograding carbonate platforms that now form the Cassian and Schlern Dolomite (Bosellini, 1998; Hausmann & Nützel, 2015; Keim et al., 2001) and can reach a thickness of over 300 m (Fürsich & Wendt, 1977). The Cassian Formation *sensu lato* includes all clay-rich Ladinian–Carnian sediments deposited in the interplatform basins of the Dolomites (Part SI). Deposition took place in the Western Tethys in a setting comparable to recent tropical environments, with warm water temperatures, seasonality, and fresh water influx (Nützel, Joachimski & Correa, 2010). Diagenetic alteration and lithification were low and fossil extraction is easy, yielding many well-preserved fossils with original skeletal microstructures and aragonite preservation (Roden et al., 2020). Many localities from this reef-basin assemblage have yielded abundant fossils, ideal for the assessment of diversity patterns and paleoecological studies (Fürsich & Wendt, 1977; Nützel, Joachimski & Correa, 2010; Roden et al., 2020). In a large quantitative study of the Cassian fauna, Fürsich & Wendt (1977) described autochthonous assemblages that are thought to represent communities. These contain recurring sets of species, which favors the idea of community-assembly processes and dynamics determining faunal heterogeneity (Drake, 1991).

**Figure 1:**
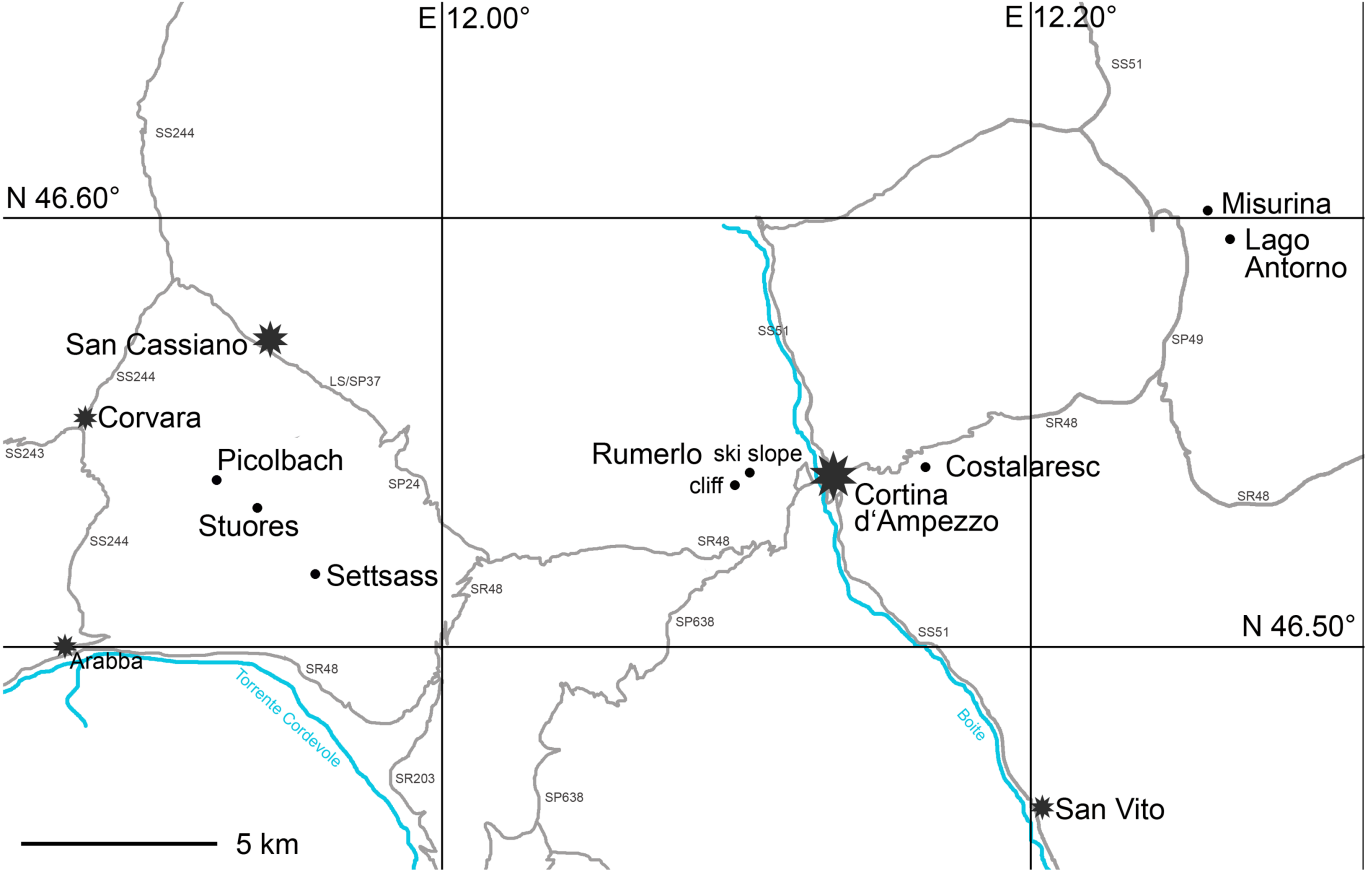
Map of studied localities in the Cassian Formation (dots) and major settlements (stars).

Surface and bulk samples from the Cassian Formation were gathered in field campaigns conducted in 2015 and 2016 (Fig. 1). Studied samples belong to the *aon, aonoides*, and *austriacum* zones, the time span that covers the vast majority of diverse benthic assemblages of the Cassian Formation *sensu lato* (Fig. S1), equivalent to approximately 5 myr. The entire formation is distributed over an area of c. 500 km^2^.

Bulk samples were disaggregated in a 7% H_2_O_2_ solution and wet-sieved with mesh sizes of 2, 1, 0.5, 0.25, and 0.125 mm. Only size fractions >0.5 mm from bulk samples are included in this study. Samples were picked and sorted using a light microscope. Roden et al. (2018) found that beta diversity, calculated as mean proportional dissimilarity, is depicted accurately when only the most abundant taxa in each sample are counted. Whereas the five most abundant species are usually sufficient, we identified and counted the ten most abundant species to avoid issues with samples of high evenness (Roden et al., 2018). All animal taxa were included, providing comparability with previously published data. In disarticulated bivalves, the few incomplete specimens were each counted as single specimens, since valves were of differing shapes and sizes. Only mollusks are among the top ten animal species in all four studied samples.

**Figure 2:**
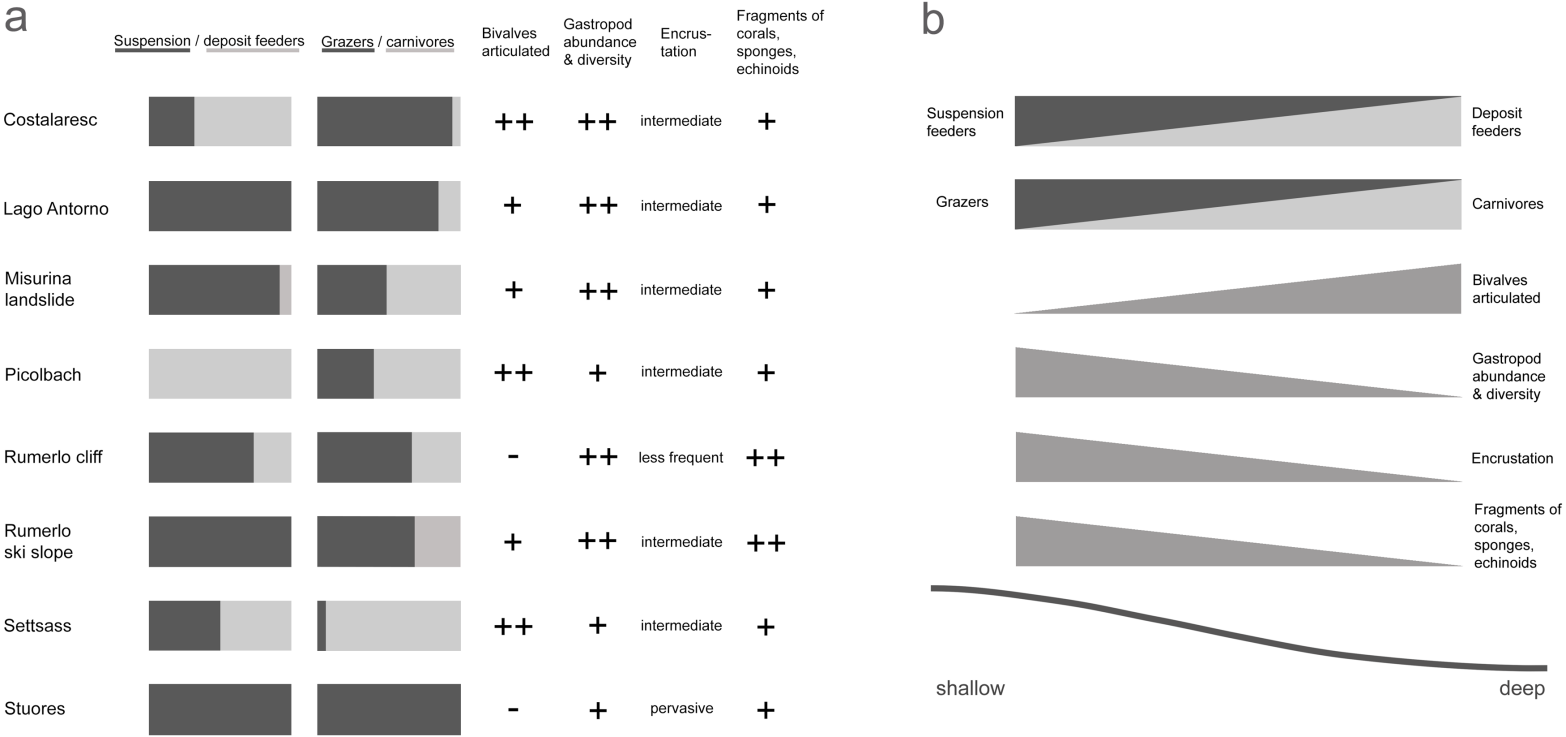
Depth-related attributes of the studied samples from the Cassian Formation (A); hypothesized changes of sample characteristics with depth (B).

We inferred paleo-water depth following the criteria of Fürsich & Wendt (1977). Inference is based on the ratio of suspension and deposit feeders, the ratio of carnivores and grazers, the proportion of articulated bivalves, the abundance and diversity of gastropods, the encrustation of specimens, and the presence of coral, sponge, and echinoderm fragments (Fig. 2). Weighting of factors is detailed in Part SII. Age, locality, and diversity of Cassian samples are provided in Table 1.

Field collection was authorized by E. Kustatscher, Museum of Nature South Tyrol. All material collected is accessioned into the collections of the Museum of Nature South Tyrol (collection numbers NMS PZO12554 to NMS PZO12598).

### Modern data

The Recent samples are from a shallow-water area in the Northern Bay of Safaga in the Red Sea, Egypt (Zuschin & Hohenegger, 1998), representing a coral-dominated, subtropical setting with warm water temperatures and seasonality, high salinity, and a highly structured bottom topography reaching down to more than 50 m water depth (Piller & Pervesler, 1989; Titschack et al., 2010). Water temperature and salinity are without any obvious depth gradient due to complete water mixing (Piller & Pervesler, 1989). Terrigenous input along with nutrients occurs mainly along the coast and is due to fluvial transport during flash floods, local erosion of impure carbonate rocks, and aeolian transport by the prevailing northerly winds (Piller & Mansour, 1994). Water energy is relatively weak, but a complex current pattern influences facies development (Piller & Pervesler, 1989). The samples were collected and processed within the scope of previous studies (Zuschin & Hohenegger, 1998; Zuschin & Oliver, 2005).

**Table 1:** Age, locality, size, and diversity of studied samples from the Cassian Formation. Samples from Lago Antorno, Misurina landslide, Settsass, and Stuores are from previous studies (Hausmann et al., 2019, unpublished data; Nützel & Kaim, 2014; Hausmann & Nützel, 2015). See ‘Methods’ for applied measures. Indices based on 10 most abundant species per sample. PPD = pairwise proportional dissimilarity. Mean PPD with regard to other samples. Inferred relative water depth is based on ranking detailed in Table S1: Positive values refer to deeper settings, negative values to shallower settings.

**Locality**	**Stratigraphic age (ammonite biozone, substage)**	**Coordinates**		**Reference**	**No. of specimens**	**Berger-Parker dominance index**	**Evenness**	**Mean PPD**	**Inferred relative water depth rank**
Costalaresc	*austriacum*, Julian	46.53995N	12.16390E	This study	315	0.73	0.29	0.91 ± 0.04	−1
Lago Antorno	probably *austriacum*, Julian	46.59438N	12.26100E	Hausmann et al. (2019, unpublished data)	216	0.31	0.50	0.88 ± 0.06	−4.5
Misurina landslide	probably *austriacum*, Julian	46.59490N	12.25962E	Hausmann et al. (2019, unpublished data)	558	0.30	0.48	0.89 ± 0.06	−2
Picolbach	*aon*, Julian	46.53427N	11.92253E	This study	213	0.28	0.54	0.92 ± 0.04	4
Rumerlo cliff	probably *aonoides*, Julian	46.53375N	12.09882E	This study	229	0.22	0.54	0.91 ± 0.04	−4
Rumerlo ski slope	*aonoides*, Julian	46.53684N	12.10214E	This study	33	0.21	0.58	0.93 ± 0.05	−5
Settsass	*aon*, ”Cordevolian”	46.51733N	11.95846E	Nützel & Kaim (2014)	296	0.24	0.50	0.91 ± 0.04	2
Stuores	*aon*, ”Cordevolian”	46.52872 N	11.93672E	Hausmann & Nützel (2015)	1026	0.33	0.53	0.95 ± 0.02	−6

Standardized bulk sampling in soft substrates was conducted at 13 sites, from shallow subtidal down to 40 m water depth (Table 2), covering an area of approximately 75 km^2^. The dataset consists only of mollusks. Whole shells >one mm were considered and disarticulated valves were counted as individuals (Zuschin & Hohenegger, 1998; Zuschin & Oliver, 2005). For comparability, the Safaga dataset was also reduced to the ten most abundant species per sample. To test whether results are robust, the complete dataset was analyzed and results are provided in Part SIII. Samples taken from the same environment, site, and depth (only several meters apart) were combined to create a by-site dataset comparable to that from the Cassian Formation. Results from the by-sample dataset are provided in Part SIV.

**Table 2:** Environment, locality, and diversity of studied samples from the Bay of Safaga. See Material and Methods for applied measures. Indices based on 10 most abundant species per sample. PPD = pairwise proportional dissimilarity. Mean PPD with regard to other samples.

**Locality**	**Environment**	**Depth (m)**	**Coordinates**		**No. of specimens**	**Berger-Parker dominance index**	**Evenness**	**Mean PPD**
94-1-a	Sand between coral patches	10	26.81417N	33.97683E	901	0.17	0.54	0.67 ± 0.11
94-1-b	Sand between coral patches	10	26.81417N	33.97683E	778	0.16	0.55	0.66 ± 0.11
94-1-c	Sand between coral patches	10	26.81417N	33.97683E	785	0.18	0.53	0.66 ± 0.11
94-1-d	Sand between coral patches	10	26.81417N	33.97683E	729	0.17	0.54	0.66 ± 0.12
94-3-a	Muddy sand	23	26.79117N	33.94667E	510	0.45	0.41	0.74 ± 0.10
94-3-b	Muddy sand	23	26.79117N	33.94667E	624	0.45	0.39	0.74 ± 0.10
94-4-a	Mud	39	26.81417N	33.96533E	2,140	0.22	0.50	0.83 ± 0.10
94-4-b	Mud	39	26.81417N	33.96533E	1,647	0.23	0.50	0.83 ± 0.10
94-5	Reef slope	19	26.84733N	34.00483E	416	0.31	0.51	0.91 ± 0.10
94-6	Mangrovechannel	<1	26.76750N	33.96283E	481	0.44	0.45	0.86 ± 0.08
95-31	Reef slope	12	26.82933N	33.98483E	1,187	0.49	0.43	0.85 ± 0.07
B-5-8	Sandy seagrass	6	26.82683N	33.95383E	2,161	0.43	0.47	0.68 ± 0.08
C-1-3	Muddy sand with seagrass	40	26.83000N	33.98683E	3,969	0.44	0.43	0.75 ± 0.09

### Diversity estimates

We assess beta diversity patterns in the Triassic Cassian Formation based on 8 bulk samples and compare the results with those from the Red Sea samples. For both datasets, we use pairwise proportional dissimilarity (relative Bray–Curtis) to compare samples. Abundance-based indices, such as proportional dissimilarity, have been shown to be relatively insensitive to uneven sample sizes, as opposed to incidence-based indices, which are biased when species richness and/or sampling completeness vary (Krebs, 1999; Magurran, 2004; Wolda, 1981). Proportional dissimilarity is calculated as d_jk_ = 1 –∑min(x_ij_, x_ik_), with x_ij_ and x _ik_ being the proportions of species abundance in each sample. Ordination plots using non-metric multidimensional scaling are also based on pairwise proportional dissimilarity. The dispersion of homogeneity is based on pairwise proportional dissimilarity as well as the modified Gower measure (Anderson, 2006; Anderson, Ellingsen & McArdle, 2006). For both beta diversity indices, the centroid for the entire dataset is figured for both the Cassian and the Safaga dataset; in addition, the sites were grouped into shallow and deeper water groups (Part SV). Community composition data from samples within a depth group are combined and a Kruskal–Wallis rank sum test is performed to compare the rank distribution of the different depth groups. Non-metric multidimensional scaling is used to visualize differences in community composition. Permutational multivariate analysis of variance (PERMANOVA) using distance matrices is applied to determine significance of depth and age groups. Calculations and visualization were implemented using R Version 3.5.0 (R Core Team, 2016) and the vegan (Oksanen et al., 2016), sads (Prado, Miranda & Chalom, 2014), and visreg (Breheny & Burchett, 2013) packages.

A null model for each dataset is created by first pooling all taxa with their observed abundances, yielding a single vector of total abundances in the dataset. From this, we derived a vector of proportional abundances. The species pool is then randomly resampled, using the proportional abundance vector as probability weights, until the number of specimens and number of sampling sites of the original datasets are obtained (e.g., until there are 8 sampling sites each containing the number of specimens as the original samples). The mean proportional dissimilarity is calculated for each simulated dataset across 1,000 iterations.

Alpha-level (local) community diversity is deduced from rank-abundance distributions of the 10 most abundant species per sample as well as the Berger-Parker dominance index (the proportion of the most abundant species) and Pielou’s evenness (J = H/log(s), where H is the Shannon index and s is the number of species) (Berger & Parker, 1970; Krebs, 1999; Pielou, 1966). Hypothesis testing was done using Spearman’s rank correlation, as pairwise dissimilarity as well as alpha diversity values are not normally distributed. Distance decay, the decrease in biological similarity with spatial distance, is measured as the correlation between pairwise proportional dissimilarity and spatial distance.

## Results

### Environments and alpha diversity

Quantitative faunal data of samples from the localities Costalaresc, Picolbach, Rumerlo cliff and Rumerlo ski slope are first reported herein (Table 3 and Figs. S14–S16). This dataset was supplemented with data from Settsass (Nützel & Kaim, 2014), Stuores (Hausmann & Nützel, 2015), and the two localities Lago Antorno and Misurina landslide (Hausmann et al., 2019, unpublished data), covering various environments (Table 1). The Stuores sample is one of the most diverse assemblages known from the Mesozoic (Hausmann & Nützel, 2015). The assemblage from Settsass is of moderate diversity and differs greatly in taxonomic composition from previously studied Cassian samples (Nützel & Kaim, 2014). The samples from Lago Antorno and Misurina landslide are relatively similar in faunal composition and are both moderately diverse (Hausmann et al., 2019, unpublished data). All samples stem from soft-bottom habitats.

**Table 3:** Faunal composition of the four new samples from the Cassian Formation. Information on the localities provided in Table 1.

Rumerlo ski slope		Costalaresc		Picolbach		Rumerlo cliff	
Species	Specimens	Species	Specimens	Species	Specimens	Species	Specimens
*Camposcala biserta*	7	*Helenostylina convexa*^c^	230	*Domerionina stuorense*	59	*Camposcala biserta*	50
*Zygopleura campoensis*	5	*Dentineritaria neritina*	18	Caenogastropoda sp. 1	24	*Ruganeritaria subovata* sensu Bandel, 2007	48
*Costactaeon* n. sp.	4	*Domerionina stuorense* sensu Nützel & Kaim (2014), Hausmann & Nützel (2015)	16	*Plagioglypta undulata*	24	*Costactaeon* n. sp.	35
*Promathildia decorata*	3	*Palaeonucula* sp. 2	10	*Palaeonucula* sp. 1	23	*Stuorilda cassiana*	30
*Fedaiella elongata* sensu Bandel, 2007	3	*Neritaria mandelslohi*	10	*Domerionina* sp. 1	22	*Zygopleura depressa*	20
*Kittliconcha?* sp.	2	*Plagioglypta undulata*	9	*Azyga dolomitensis*	15	*Tofanella cancellata*	9
*Zygopleura hybridissima*	2	*Ampezzopleura hybridopsis*	7	*Domerionina pralongiana*	15	*Teretrina* cf. *bolina*	9
*Ampezzopleura bandeli*	2	*Atorcula anoptychopsis*	5	*Helenostylina convexa*^c^	11	*Prostylifer paludinaris*	7
*Neritaria plicatilis*	2	*Ampezzopleura bandeli*	4	*Stuorilda cassiana*	10	*Coelostylina conica*	6
*Popenella misurina, Stuorilda tichyi, Rinaldoconchus bieleri^a^*	1	*Spirostylus brevior, Domerionina* sp. 1^b^	3	*Neritaria mandelslohi*	10	*Domerionina* n. sp., *Palaeonucula* sp. 1, *Frederikella cancellata*^d^	5

**Notes.**

After excluding foraminifers from one sample (Rumerlo ski slope, 11 specimens of *Pragsoconulus robustus*) for comparability (the four samples from earlier studies did not consider foraminifers), the dataset only contains mollusks. Occurrences of brachiopods, sponges, corals, and echinoderms contained in some samples were not among the 10 most abundant species and are not reported here.

a Each represented with one specimen. Rumerlo ski slope yielded a very low number of specimens.

b Each represented with 3 specimens.

c *Helenostylina convexa* is identical with *Ptychostoma sanctaecrucis* as figured by Bandel (1992, pl. 6, figs. 4 and 5).

d Each represented with 5 specimens.

**Figure 3:**
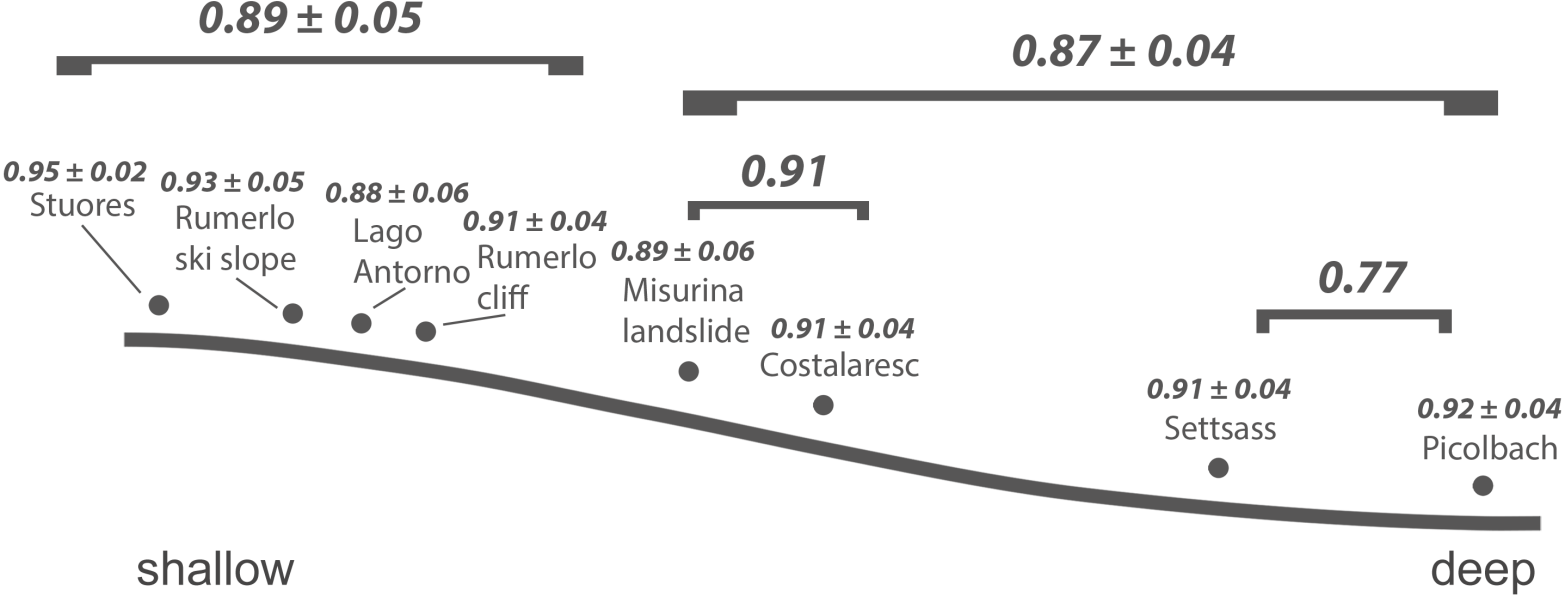
Inferred bathymetric gradient of the Cassian samples. Mean pairwise proportional dissimilarity (PPD) of each sample with other samples as well as mean PPD among samples or PPD between samples grouped by depth are depicted. Ranges refer to one standard error.

The samples are arranged along a shallow reefal to basinal transect (Fig. 3) based on inferred water depth (Table S1). The bathymetric partitioning among the Cassian localities is only relative, rendering a comparison with recent settings difficult. There were no apparent differences in the sediment matrix among samples. Due to the high proportion of grazers and high gastropod abundance and diversity, we describe Costalaresc as originating from a relatively shallow environment, but—with a high proportion of deposit feeders and all specimens of bivalves being articulated—we interpret a slightly deeper setting than for the Rumerlo localities. Rumerlo ski slope is interpreted as a very proximal back reef setting, due to the large number of fragments of corals, sponges, and echinoids, among other factors (see Part SII). Picolbach probably stems from a deeper setting, as interpreted from mode of life of reported specimens and articulated bivalves. Evenness values, Berger-Parker dominance index (Table 1), and rank-abundance distributions (Fig. S12, Part SVI) demonstrate relatively diverse assemblages, with the exception of Costalaresc. Low alpha diversity in Costalaresc is due to the dominance of the gastropod *Helenostylina convexa*. There is no significant correlation between inferred water depth and alpha diversity measured as dominance (Spearman’s rho = 0.02, *p* = 0.98) or evenness (rho = 0.29, *p* = 0.50) in the Cassian samples.

Environments and depth from which the Safaga samples were taken are recorded and alpha diversity calculated (Table 2). Samples from the reef slope and from sand between coral patches show lowest dominance and highest evenness. There is no significant correlation between alpha diversity and depth in the Safaga samples (dominance: rho = −0.05, *p* = 0.93, evenness: rho = −0.12, *p* = 0.78). The range of evenness is slightly higher (*J* = 0.39 to 0.55) than in the Cassian samples (*J* = 0.29 to 0.58).

### Beta diversity

**Figure 4:**
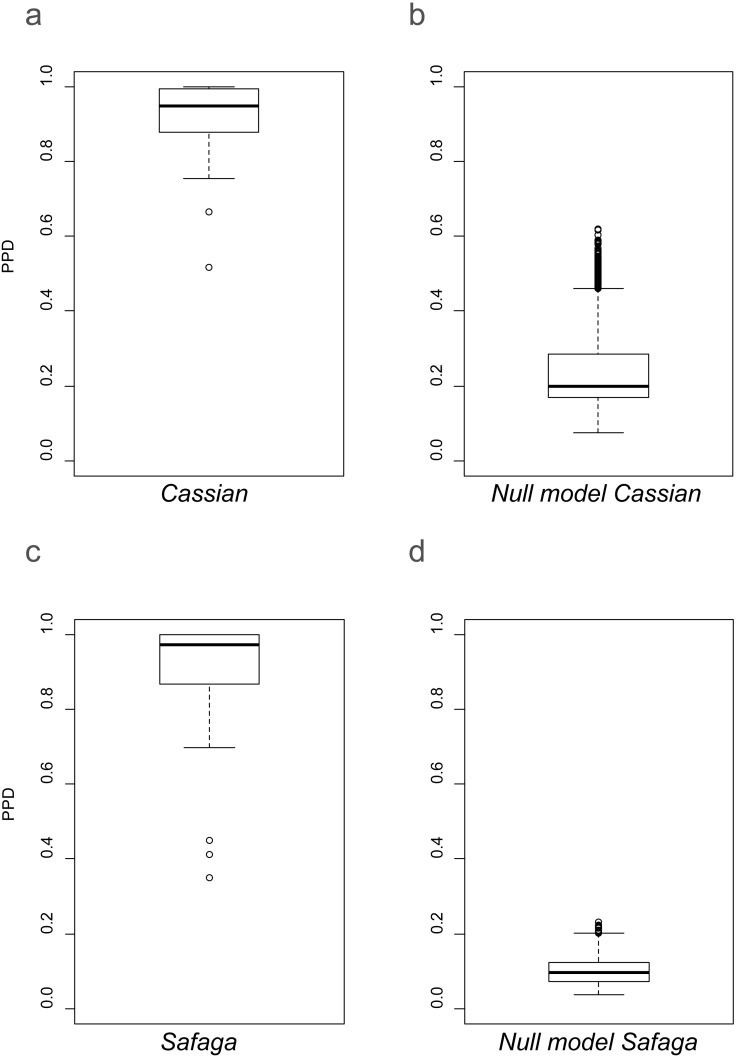
Beta diversity as pairwise proportional dissimilarity (PPD) in observed (A, C) and permuted (B, D) datasets. Observed PPD of the Cassian (A) and the by-site Safaga (C) datasets. Distribution of mean PPD of the null model based on the Cassian dataset (B) and the by-site Safaga dataset (d). Mean PPD for the null models were calculated over 1,000 iterations.

**Figure 5:**
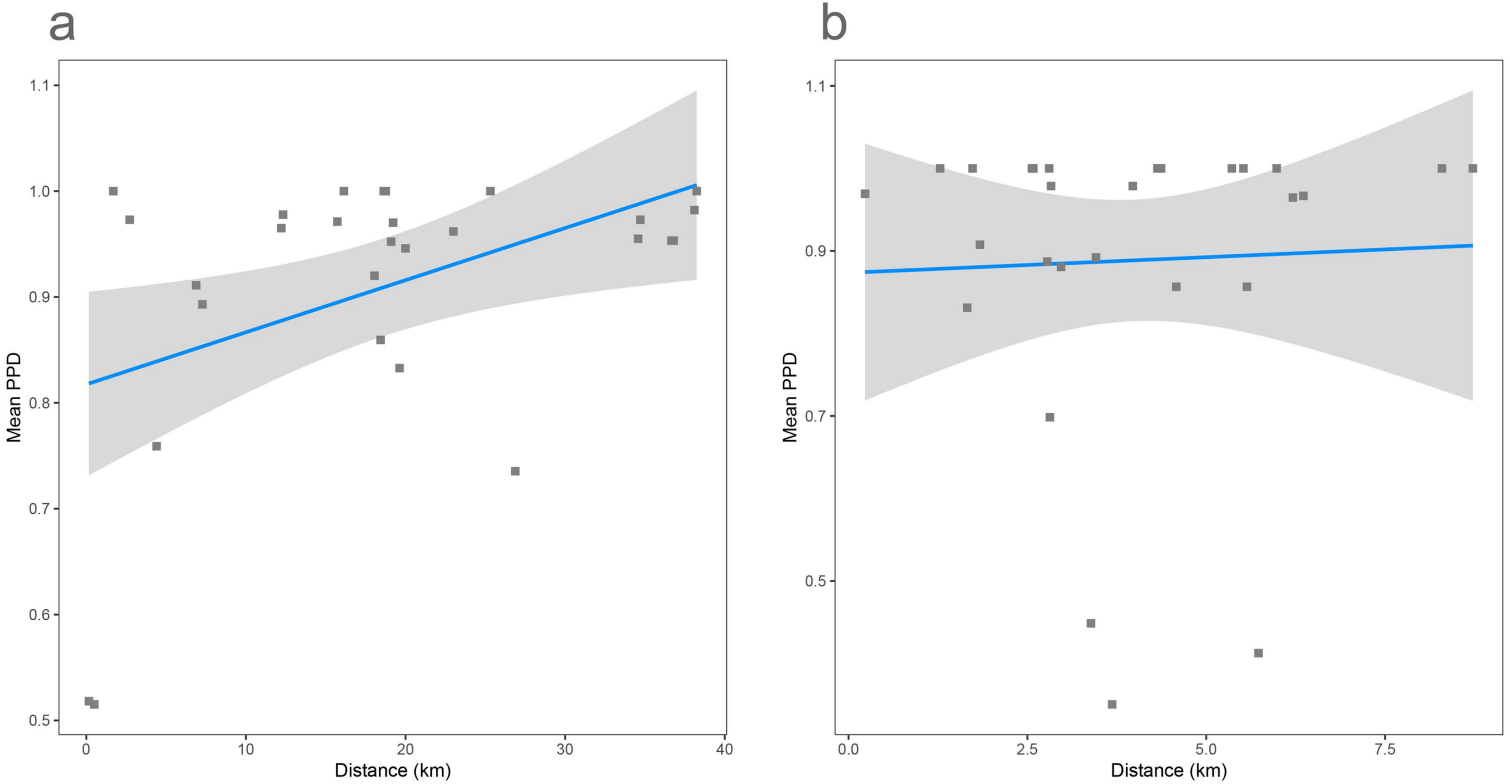
Distance decay calculated as correlation between spatial distance (based on the GPS coordinates provided in Tables 1 and 2) and pairwise proportional dissimilarity in the Cassian dataset (A) and the by-site Safaga dataset (B). Distance decay is non-significant for the Cassian when using ranks (Spearman’s rank correlation rho = 0.14, *p* = 0.48) and when using Pearson’s product-moment correlation (Pearson correlation = 0.38, *p* = 0.055; however, values are not normally distributed). Distance decay is non-significant for the Safaga dataset (Pearson correlation = 0.05, *p* = 0.83, rho = 0.05, *p* = 0.81).

As shown previously, overall beta diversity is high in the Cassian Formation (mean PPD: 0.91 ± 0.02, ± is standard error; range: 0.52–1; Table 4). In the by-site dataset from Safaga, we measure a beta diversity of 0.89 ±  0.04 (range: 0.35–1.00; Table 5). Null models created for each dataset from the gamma species pool yield much lower beta diversity, with a mean of 0.24 ± 0.0004 for the Cassian dataset (Fig. 4A) and 0.10 ± 0.0002 for the by-site Safaga dataset (Fig. 4B). Histograms show the distribution of pairwise dissimilarity values (Fig. S13). There is no significant correlation between spatial distance and dissimilarity neither in the Cassian samples (Spearman’s rho = 0.14, *p* = 0.48; Fig. 5A) nor the by-site Safaga dataset (rho = 0.05, *p* = 0.81; Fig. 5B).

**Table 4:** Pairwise proportional dissimilarity (PPD) of the studied samples from the Cassian Formation. Information on the localities provided in Table 1. PPD based on 10 most abundant species per sample.

	**Lago Antorno**	**Misurina landslide**	**Picolbach**	**Rumerlo cliff**	**Rumerlo ski slope**	**Settsass**	**Stuores**
Costalaresc	0.86	0.91	0.83	1.00	0.99	0.76	1.00
Lago Antorno		0.52	0.93	0.94	1.00	0.95	0.95
Misurina landslide			1.00	0.94	0.96	0.96	0.95
Picolbach				0.93	1.00	0.77	1.00
Rumerlo cliff					0.67	0.97	0.89
Rumerlo ski slope						1.00	0.86
Settsass							0.97

**Table 5:** Pairwise proportional dissimilarity (PPD) of the studied samples from the Bay of Safaga. Samples taken from same site (only several meters apart) were combined (94-1-a to -d: 94-1, 94-3-a and -b: 94-3, 94-4-a and -b: 94-4-a). Information on the localities provided in Table 2. PPD based on 10 most abundant species per sample.

		**94-1**	**94-3**	**94-4**	**94-5**	**94-6**	**95-31**	** B-5-8**	** C-1-3**
		Sand between coral patches	Muddy sand	Mud	Reef slope	Mangrove channel	Reef slope	Sandy seagrass	Muddy sand with seagrass
94-1	Sand between coral patches		0.97	1.00	1.00	0.86	0.83	0.70	0.91
94-3	Muddy sand			0.88	1.00	0.97	0.99	0.44	0.42
94-4	Mud				1.00	1.00	1.00	1.00	1.00
94-5	Reef slope					1.00	0.89	1.00	1.00
94-6	Mangrove channel						0.96	0.86	0.97
95-31	Reef slope							0.89	0.97
B-5-8	Sandy seagrass								0.35

**Figure 6:**
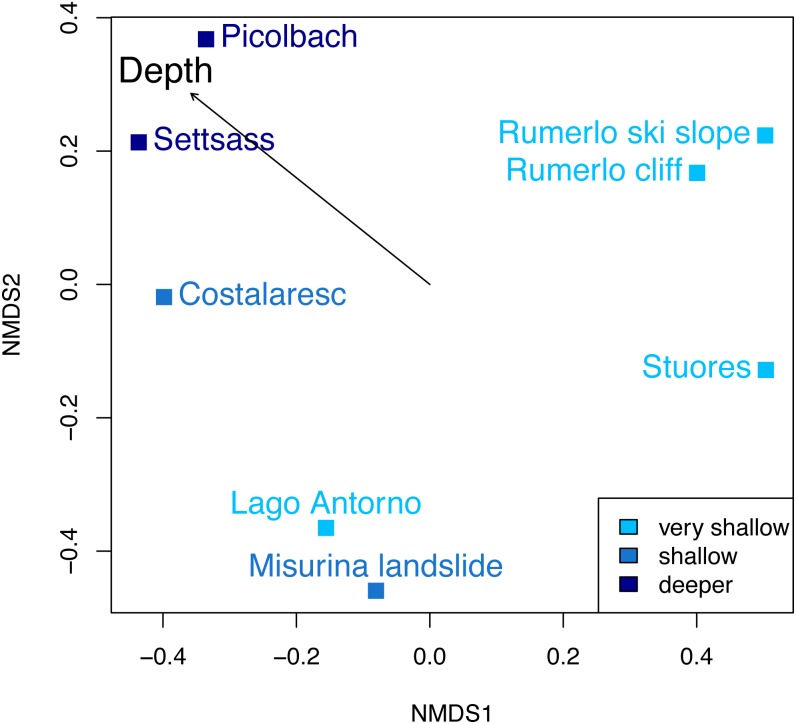
Non-metric multidimensional scaling of the Cassian samples, color-coded by depth. There are no distinct associations among localities from similar depths. Stress value is 0.06. Depth clusters are non-significant (*R*^2^ = 0.23, *p* = 0.06). Arrow represents fitting of environmental factor depth as bathymetric gradient.

Dissimilarity between depth categories does not differ much from dissimilarity within depth groups in either dataset. Grouping Cassian samples by depth yields a dissimilarity of 0.83 between the four shallower and the four deeper localities (see Table 1 and Table S1). Using three depth categories (the four shallowest localities, two intermediate and two deeper localities, see also Fig. 3), we find the following dissimilarities: shallow/intermediate: PPD = 0.83, intermediate/deep: PPD = 0.78, shallow/deep: PPD = 0.94). Dissimilarity between the two localities from deeper environments is lower than among the shallower localities, while the two sites from an intermediate depth are very dissimilar (Fig. 3). Non-metric multidimensional scaling shows no clear distinction between localities based on depth (Fig. 6). Neither two nor three depth groups are statistically supported by PERMANOVA (*R*^2^ = 0.21, *p* = 0.15; *R*^2^ = 0.23, *p* = 0.06, respectively). The Cassian data show relatively broadly dispersed sites when plotting the dispersion of homogeneity of all assemblages. Differentiating between shallow- and deeper-water assemblages yields two separate groups for PPD but overlapping centroids when the modified Gower measure is applied (Fig. S10 , Part SV). Kruskal–Wallis rank sum tests show that the shallow- and deeper-water assemblages have statistically indistinguishable distributions (*p* = 0.07). Using three groups, rank sum tests also yield the same rank distributions (shallow/deep: *p* = 0.07, shallow/intermediate: *p* = 0.31, shallow/deep: *p* = 0.06). There is no correlation of paleo-depth with mean PPD of each site (Spearman’s rho = 0.24, *p* = 0.58). Temporal turnover between the ammonite biozones is only slightly higher than dissimilarity within the ammonite biozones, which strongly varies (Part SI). We find age to explain 43% of the variation in community dissimilarity (*p* = 0.02).

**Figure 7:**
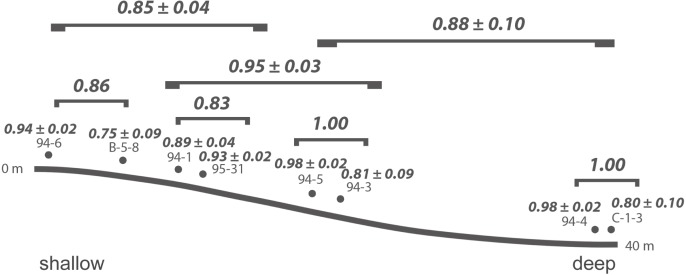
Mean PPD among samples and depth categories for the by-site Safaga dataset.

**Figure 8:**
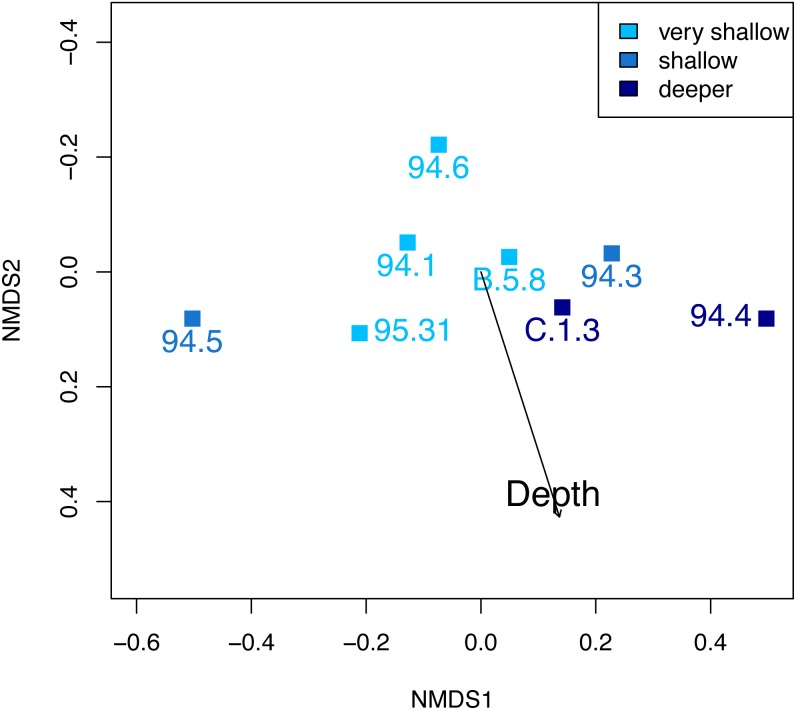
Non-metric multidimensional scaling of the Safaga samples, color-coded by depth. As in the Cassian (Fig. 6), there are no distinct associations between localities from similar depths. Stress value is 0.01. Depth clusters are non-significant (*R*^2^ = 0.17, *p* = 0.28).

At Safaga, there is also no clear relationship between beta diversity and water depth (Fig. 7). As in Cassian, there is no distinct association of samples from similar water depths at Safaga (Fig. 8). Grouping samples into two or three depth groups yields non-significant clusters (*R*^2^ = 0.17, *p* = 0.28). Dispersion of homogeneity yields similar results as in the Cassian dataset (Fig. S11). Samples taken from deep, muddy settings exhibit the highest mean beta diversity relative to other samples (0.98 ±  0.02). Otherwise, there is no relationship between sedimentary attributes and mean dissimilarity. By grouping the samples into depth ranges, we cover several environments for each. Samples from shallower environments have a more similar community composition than samples from deeper environments (Figs. 7 and 8). There is no correlation of water depth with mean PPD of each site (Spearman’s rho = 0.02, *p* = 0.98). Dissimilarity between the four shallower and the four deeper samples is 0.77 and therefore lower than other values measured within depth ranges. Using three depth categories (the four shallowest localities, two intermediate and two deeper localities), we find the following dissimilarities: shallow/intermediate: PPD = 0.79, intermediate/deep: PPD = 0.60, shallow/deep: PPD = 0.77). In contrast to the Cassian data, Kruskal–Wallis rank sum tests show that all depth groups have different rank distributions (*p* <  10^−16^) in the Safaga dataset.

## Discussion

### Environment

We find no distinct trends in alpha diversity with depth in either dataset. Measures of evenness and dominance show relatively high alpha diversity for most samples in both datasets, from all environments and depths. Low dominance is corroborated by another study on soft-bottom taxa in the central Red Sea (Alsaffar et al., 2019). Patterns of a bathymetric diversity gradient in benthic fauna in the literature are conflicting (e.g., Brown & Thatje, 2014; Gray et al., 1997; Holte, Oug & Cochrane, 2004; Martins, Quintino & Rodrigues, 2013).

Beta diversity has not been studied extensively in soft-bottom habitats, with only a few studies in non-reefal soft-bottom settings (e.g., Aldea, Olabarria & Troncoso, 2009; Ellingsen, 2002; Koulouri et al., 2006). Our study shows high beta diversity in two reef-associated soft-bottom assemblages from warm-water settings. We find no significant contributions of water depth, spatial distance, and stochastic processes to beta diversity. There is a moderate influence of age in the Cassian samples.

Time averaging generally lowers beta diversity in an assemblage (Tomašových & Kidwell, 2009). However, we can assume that the individual assemblages in the Cassian are time-averaged to a similar degree as in Safaga (i.e., within the same habitat over hundreds to few thousands of years). But although time averaging does not hamper the comparison, the differing temporal ranges may (5 myr vs. <1,000 years, respectively). High beta diversity within ammonite biozones in the Cassian points to a true ecological signal, but unfortunately, we do not know what temporal range lies between two samples from the same biozone. Temporal differences between some samples are in the range of millions of years.

The high beta diversity in the Cassian Formation is remarkable, considering that all samples stem from soft-bottom habitats adjacent to reefs—most likely a more uniform habitat than the reef structure. The slightly lower beta diversity of modern Safaga compared with ancient Cassian is surprising considering that certain environments such as mangrove and seagrass, which contribute to beta diversity at Safaga, did not yet exist in the Triassic period (Hemminga & Duarte, 2000; Ricklefs, Schwarzbach & Renner, 2006). In addition, the bottom topography of Safaga is highly structured for a soft-bottom habitat (Zuschin & Oliver, 2005).

Results from grouping the Cassian localities by inferred depth are ambiguous. While dissimilarity between the two localities from deeper environments is lower than most other values, data are too limited to allow for robust conclusions on beta diversity vs. depth. However, the Safaga samples—with a larger number of sampling sites and specific recorded depths—yield slightly higher beta diversity in deeper habitats. In addition, dissimilarity between two depth categories (four shallower samples vs. four deeper samples) is lower than within depth categories for both datasets. The high beta diversity at both Cassian and Safaga may partly be driven by the great range of environments included. Specifically, beta diversity has been shown to increase with the variance of depths, due to the larger range of communities included in the study (Harborne et al., 2006).

Change in faunal composition with depth is usually linked to variations in environmental factors, such as temperature, salinity, oxygenation, or sediment characteristics (Durden et al., 2015; Ellingsen & Gray, 2002; Holte, Oug & Cochrane, 2004; Laine, 2003). Due to water mixing in the Bay of Safaga (Zuschin & Oliver, 2005), there is probably no depth gradient related to temperature, salinity, or oxygenation, but light penetration may play a role. Differences in sedimentary attributes are not related to faunal dissimilarity. While samples from a muddy environment have the highest mean dissimilarity with the other samples from Safaga (see also ordination in Zuschin & Hohenegger, 1998: fig. 11a), this may be related to other factors, as there is no trend in beta diversity from sand to muddy sand to mud. Muddy habitat is common in the Red Sea in deeper waters and occurs in a shallower settings in protected depressions. Especially the very high dissimilarity between samples from similar depths at Safaga and in the Cassian contradicts findings by Ellingsen & Gray (2002) that samples from similar depths show higher similarity. While the Safaga samples differ in habitat and grain size, differences in sedimentary attributes were not noted in the Cassian Formation, leading us to assume that other factors may play a larger role in determining faunal composition. While depth is often found to be an important driver of beta diversity (Ellingsen & Gray, 2002; Harborne et al., 2006), we find no changes in beta diversity with depth in our sites. However, depth can determine faunal composition through depth-range restriction of species (Rex & Etter, 2010), which may explain high overall beta diversity when differences in water depth are large.

Comparing dissimilarity among different datasets is made more difficult by the fact that spatial resolution influences beta diversity. Small spatial scales yield higher beta diversity than larger scales in coral reefs and rain forests, as the heterogeneity of environments is limited (Pandolfi, 2002). However, studies on the effects of spatial scale on beta diversity reach differing conclusions (Lennon et al., 2001). Specifically, small-scale environmental differences—often created by the biota itself—can increase faunal variability. These small-scale differences can in turn increase beta diversity at a larger scale (e.g., Thrush, Lundquist & Hewitt, 2005). Applying Pandolfi’s (2002) three-phase model of variability to the reef-adjacent soft bottoms of the Cassian Formation and the Bay of Safaga, we find that variance in community composition is expected to be lower at the observed spatial extent (1–100 km distance) of both datasets than at smaller (<1 km) or larger (>1,000 km) scales. We therefore conclude that the high values of beta diversity are genuine and not due to spatial scale.

### Stochasticity and distance decay

The lack of correlation between spatial distance and community dissimilarity in the two sites lets us assume that there is no noteworthy distance decay over the limited spatial extent covered by the fossil and modern datasets. Studying an environmentally very homogeneous soft-bottom habitat on the Norwegian continental shelf, Ellingsen (2002) also found a weak relationship between spatial distance and dissimilarity. In addition, beta diversity in both study sites is much higher than predicted from the null models. Therefore, the spatial patterns of community composition among the study sites are not random and probably not constrained by dispersal. Since the two datasets differ in size (the Safaga dataset contains a higher number of samples, specimens, and species over a smaller area), the higher beta diversity in the Cassian null model is probably due to increased randomness by sampling fewer specimens and species from a slightly smaller species pool. With a beta diversity of 0.91 for the Cassian Formation despite the small species pool, stochasticity is clearly not the main driver of beta diversity. With the lack of stochasticity on top of the lack of a spatial pattern related to distance, other factors must have a stronger control on beta diversity.

### Community assembly

Although we find no significant change in beta diversity with water depth, the factors driving beta diversity may vary with depth. In shallower settings, physical disturbances, such as storms, may have a large impact on benthic communities. In greater depths, competition may be a stronger driving force due to less prevalent disturbances and predation (Harper & Peck, 2016; Klompmaker & Finnegan, 2018). Klompmaker & Finnegan (2018) hypothesize predation to be an important factor in structuring modern and fossil soft-bottom communities, supporting earlier assessments (Stanley, 2008). However, predation has increased since the Triassic (Huntley & Kowalewski, 2007), therefore the Cassian fauna might have been less affected by predation than the Safaga fauna.

Environmental heterogeneity is often considered the main driver of faunal heterogeneity (e.g., Carlos-Júnior et al., 2019; Ellingsen, 2002; Pandolfi & Jackson, 2001; Stegen et al., 2013), with more uniform environments generally yielding a more homogeneous fauna (e.g., Clarke & Lidgard, 2000; Ellingsen & Gray, 2002; Fagerstrom, 1983; Shin & Ellingsen, 2004). However, it is often ignored that the environment can be structured by the biota itself. Organisms with hard parts that live on the sediment are found to directly contribute to environmental heterogeneity and in turn to faunal heterogeneity (Hewitt et al., 2005). The studied assemblages include both epifauna and infauna, which may directly contribute to high beta diversity through an increase in diversity driven by the combination of higher sediment stability in infaunal assemblages and biotic interactions in epifaunal assemblages (Van der Zee et al., 2015).

Besides structuring of the seafloor by the biota, we presume priority effects may be one of the drivers of beta diversity in the modern and ancient reef-related soft-bottom habitats. While there are many mechanisms that lead to differences in community composition, the order in which organisms settle in a habitat directly affects the community that subsequently assembles (Drake, 1991; Fukami, 2015; Thrush, Lundquist & Hewitt, 2005). Priority effects influence community composition at smaller sites by affecting the regional species pool as well as local population dynamics (Fukami, 2015). In addition, the first species to arrive may even gain an evolutionary advantage, as they adapt to environmental conditions sooner than subsequent arrivals (De Meester et al., 2016). Further research may reveal one or both of these effects as drivers of beta diversity. Our results show that water depth, stratigraphic age, geographic distance, and random dispersal are not the key determinants of beta diversity in the studied reef basin assemblages.

## Conclusions

Large variations in community composition are evident in reef-associated soft-bottom assemblages such as the Triassic Cassian Formation and the modern Bay of Safaga. Our original hypothesis that beta diversity decreases with depth is not supported by our analyses. We find depth, sediment structure, and stochastic effects to not significantly contribute in determining beta diversity. Temporal turnover plays a moderate role in the Cassian dataset but does not explain high beta diversity within ammonite biozones. Through exclusion of other drivers, we presume structuring of the sediment by the biota and/or priority effects to play a key role in determining community structure for both assemblages.

##  Supplemental Information

10.7717/peerj.9139/supp-1 Supplemental Information 1 Raw data

10.7717/peerj.9139/supp-2 Supplemental Information 2 Code for analyses

10.7717/peerj.9139/supp-3 Supplemental Information 3 Supplementary information

10.7717/peerj.9139/supp-4 Supplemental Information 4 Collection numbers

## Additional Information and Declarations

### Field Study Permissions

The following information was supplied relating to field study approvals (i.e., approving body and any reference numbers): Field work in the Dolomites was conducted with permission from PD Dr. Evelyn Kustatscher, Museum of Nature South Tyrol.

### Data Availability

The following information was supplied regarding data availability: Raw data, R code, a list of collection numbers, and additional information are available in the Supplementary Files. All material collected is accessioned into the collections of the Museum of Nature South Tyrol (collection numbers NMS PZO12554 to NMS PZO12598).
