# Supplementary material for: Drivers of beta diversity in modern and ancient reef-associated soft-bottom environments
Source: PeerJ. 2020 May 14;8:e9139. doi: 10.7717/peerj.9139 (PMC7231500; doi:10.7717/peerj.9139)
Supplement: Supplemental Information 3 [file peerj-08-9139-s003.pdf]

Supplementary information to

Roden VJ, Zuschin M, Nützel A, Hausmann IM and Kiessling W. Drivers of beta diversity in modern and ancient reef-associated soft-bottom environments. *PeerJ.*

Part I: Temporal changes

Herein, we apply the definition of the Cassian Formation as *sensu lato*, in a traditional sense, including all marly or clayey Ladinian–Carnian sediments deposited in the interplatform basins of the Dolomites (Fig. S1) (Bizzarini & Laghi 2005; Keim et al. 2006; Nose et al. 2018; Urlichs 2017). Studied samples are from the *aon, aonoides*, and *austriacum* zones. The base of the *aon* Zone is at 236 Ma and the top of the *austriacum* zone is at 231 Ma (Gradstein et al. 2012), covering a temporal range of approximately 5 Ma.
Temporal turnover, calculated by pooling the faunal communities of each ammonite biozone, is also relatively high (PPD *austriacum*/*aonoides* zones: 0.94, *aonoides*/*aon* zones: 0.89). Dissimilarity between the two localities from the *aonoides* zone is lower (PPD: 0.67) than among *austriacum* (mean PPD: 0.76 ± 0.12) localities and *aon* (mean PPD: 0.91 ± 0.07) localities. A visualization of community dissimilarity using NMDS shows a moderate relationship among some localities from the same ammonite biozones (Fig. S2). Testing these relationships, we found stratigraphic age to explain 43% of the variation ($p = 0.02$). However, while we do report temporal turnover in the Cassian Formation, beta diversity within the ammonite biozones is also high. Therefore, we conclude time to play a moderate role in the observed community dissimilarities. To detail the relationship between community composition and age, ranges of species are plotted in Fig. S3.

<table>
<tr><td rowspan="5">Late Triassic</td><td rowspan="5">Carnian</td><td rowspan="7">Cassian Formation sensu lato</td><td rowspan="2">Heiligkreuz Formation</td><td>Tuvalian</td><td>dilleri zone</td></tr>
<tr><td rowspan="2">Julian</td><td>austriacum zone</td></tr>
<tr><td rowspan="4">Cassian Formation sensu stricto</td><td>aonoides zone</td></tr>
<tr><td rowspan="2">"Cordevolian"</td><td>aon zone</td></tr>
<tr><td>canadensis zone</td></tr>
<tr><td rowspan="2"></td><td rowspan="2">Ladinian</td><td rowspan="2">Longobardian</td><td rowspan="2">regoledanus zone</td></tr>
<tr><td></td></tr>
</table>

Fig. S1: Stratigraphy of the Cassian Formation with ammonite zonation. Studied samples from marked ammonite biozones. The Cassian Formation *sensu stricto* comprises the late *regoledanus, canadensis, aon,* and *aonoides* ammonite biozones. The *austriacum* and *dilleri* ammonite biozones are now regarded as belonging to the Heiligkreuz Formation.

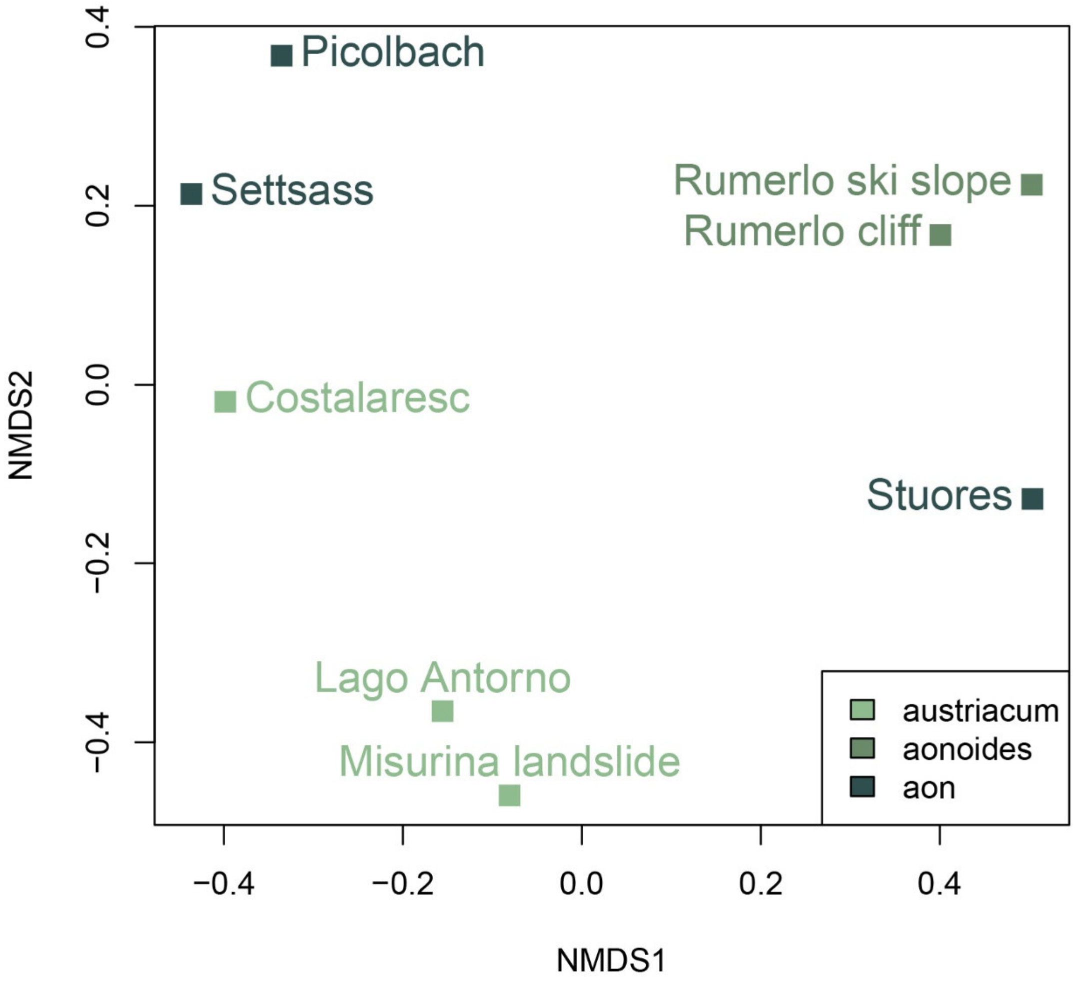

Fig. S2: Non-metric multidimensional scaling of the Cassian samples, color-coded by ammonite biozone (darker colors represent older strata). Stress value is 0.06.

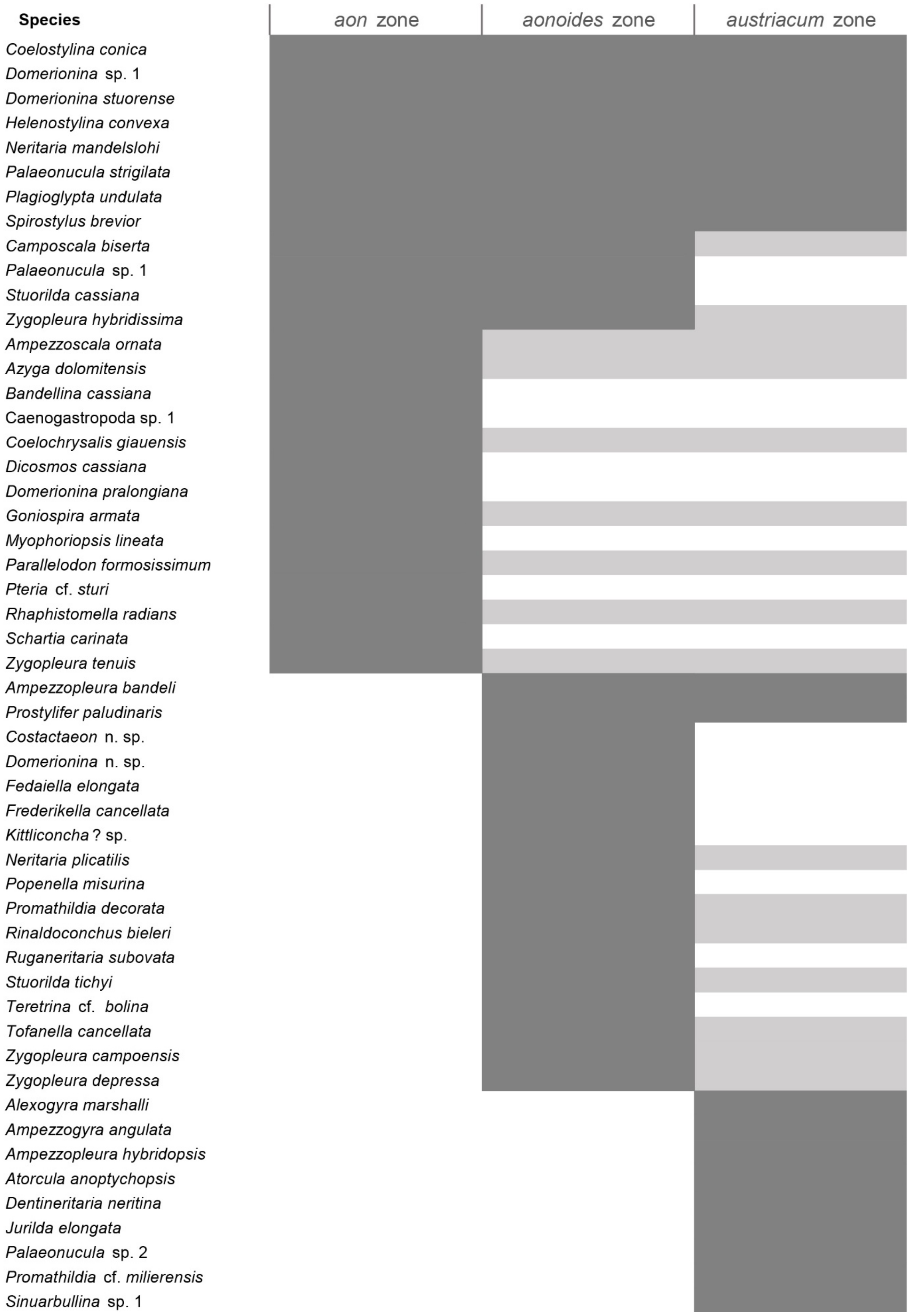

Fig. S3: Temporal ranges of species from this study. Species occurring in earliest (*aon*) and latest (*austriacum*) zone are assumed to occur in the intermediate (*aonoides*) zone (range-through assumption). Dark grey represents occurrences from this study, light grey represents additional known occurrences from the literature (based on Roden et al. 2019, appendix).

Part II: Inferred water depth of the Cassian samples

Each of the criteria from Fürsich & Wendt (1977) was rated and the values summed to rank water depth among the samples. Positive values refer to factors predicting an origin among deeper water depths, negative values refer to a shallower setting. The following factors were analyzed semi-quantitatively, the range of possible values is provided in parentheses: the ratio of suspension to deposit feeders (-2 to 2), the ratio of carnivores to grazers (-2 to 2), the proportion of articulated bivalves (-1 to 1), the abundance and diversity of gastropods (-1 to 1), the encrustation of specimens (-1 to 1), and the presence of coral, sponge, and echinoderm fragments (-1 to 1). Table S1 shows the inferred values and subsequent ranking along the reef basin.

Table S1: Factors used to infer water depth of Cassian samples. Positive values refer to deeper settings, negative values to shallower settings. The last column (*Total*) yields subsequent ranking. See Fig. 2 in main text.

	**Suspension : deposit feeders**	**Grazers : carnivores**	**Bivalves articulated**	**Gastropod abundance and diversity**	**Encrustation**	**Fragments of corals, sponges, echinoids**	**Total**
**Costalaresc**	1	-2	1	-1	0	0	**-1**
**Lago Antorno**	-2	-1.5	0	-1	0	0	**-4.5**
**Misurina landslide**	-1	0	0	-1	0	0	**-2**
**Picolbach**	2	1	1	0	0	0	**4**
**Rumerlo cliff**	-1	-1	-1	-1	1	-1	**-4**
**Rumerlo ski slope**	-2	-1	0	-1	0	-1	**-5**
**Settsass**	0	1	1	0	0	0	**2**
**Stuores**	-2	-2	-1	0	-1	0	**-6**

Part III: Reduced versus complete dataset, Bay of Safaga

The Safaga dataset was reduced to the ten most abundant species for comparability with the Cassian dataset. To test whether results are robust, the analyses using the complete Safaga dataset are provided here.

RESULTS

Environments

Environments and depth from which the Safaga samples were taken are recorded and alpha diversity calculated (Table S2). Samples from the reef slope and from sand between coral patches show highest evenness; samples from sand between coral patches show lowest dominance. There is also no significant correlation between alpha diversity, either measured as dominance or evenness, and depth in the Safaga samples. The range of evenness is slightly higher than in the Cassian samples.

Beta diversity

Overall beta diversity of the modern Safaga dataset is lower (0.80 ± 0.03; range: 0.09-0.99; Table S3) than in the Triassic Cassian Formation. When samples taken from the same site in Safaga Bay are combined (=by-site dataset), we measure a beta diversity of 0.85 ± 0.03. Null models created for each dataset from the gamma species pool yield much lower beta diversity, with a mean of 0.18 ± 0.0001 for the by-site Safaga dataset (8 samples) and 0.20 ± 0.0001 for the by-sample dataset (13 samples) (Fig. S4).

The Safaga dataset shows no distance decay (rho: 0.13, p-value: 0.49; Fig. S5a) when samples taken from the same sites are pooled (=by-site dataset). Without pooling (=by-sample dataset), there is a low correlation (rho: 0.28, p-value: 0.013; Fig. S5b) in the Safaga samples.

At Safaga, there is no clear pattern of beta diversity related to depth (Fig. S6). Samples taken from the same environment, locality, and depth (only several meters apart) are very similar. Samples taken from deep, muddy settings exhibit the highest mean beta diversity (0.95 ± 0.02). Otherwise there is no relationship between sedimentary attributes and mean dissimilarity. By grouping the samples into depth ranges, we cover several environments for each range. Grouping the localities into two, three, or four depth ranges, we generally find that samples from shallower environments have a slightly more similar community composition than samples from deeper environments (Fig. S6). Dissimilarity between the four shallower and the four deeper samples is 0.72 and therefore lower than other values measured within depth ranges. A visualization of community dissimilarity using NMDS does not show strong relationships among localities from similar depths (Fig. S7).

CONCLUSIONS

Overall beta diversity of the complete dataset (0.80 by-sample, 0.85 by-site) is slightly lower than beta diversity for the dataset containing only the ten most abundant species per sample (0.82 by-sample, 0.89 by-site). This corroborates results from Roden et al. (2018), who found beta diversity estimates based on the five or ten most abundant species per sample to be statistically indistinguishable from estimates using the complete dataset. Beta diversity for most datasets – including the same dataset from the Bay of Safaga – is only slightly higher when only abundant species are included. Null models yield higher beta diversity for the complete dataset.

Since the two Safaga datasets differ in size (the complete dataset contains 23 190 specimens and 639 species, the reduced dataset contains 16 328 specimens and 59 species), the higher beta diversity in the Cassian null model (2901 specimens and 50 species) is probably due to increased randomness by

sampling fewer specimens from a smaller species pool. Other results (distance decay, mean PPD vs. depth, NMDS) are very close to results from the reduced dataset. Therefore, overall results are robust.

Table S2: Environment, locality, and diversity of studied samples from the Bay of Safaga. See *Material and Methods* for applied measures. PPD = pairwise proportional dissimilarity. Mean PPD with regard to other samples.

**Locality**	**Environment**	**Depth**	**Coordinates**		**No. of specimens**	**Berger-Parker Dominance Index**	**Evenness**	**Mean PPD**
**94-1-a**	Sand between coral patches	10	26.81417 N	33.97683 E	1637	0.09	0.61	0.70 ± 0.08
**94-1-b**	Sand between coral patches	10	26.81417 N	33.97683 E	1454	0.09	0.62	0.70 ± 0.08
**94-1-c**	Sand between coral patches	10	26.81417 N	33.97683 E	1283	0.11	0.57	0.71 ± 0.08
**94-1-d**	Sand between coral patches	10	26.81417 N	33.97683 E	1311	0.10	0.60	0.70 ± 0.08
**94-3-a**	Muddy sand	23	26.79117 N	33.94667 E	651	0.35	0.41	0.79 ± 0.07
**94-3-b**	Muddy sand	23	26.79117 N	33.94667 E	767	0.36	0.38	0.78 ± 0.07
**94-4-a**	Mud	39	26.81417 N	33.96533 E	2353	0.20	0.38	0.87 ± 0.07
**94-4-b**	Mud	39	26.81417 N	33.96533 E	1901	0.20	0.41	0.87 ± 0.07
**94-5**	Reef slope	19	26.84733 N	34.00483 E	877	0.15	0.63	0.93 ± 0.03
**94-6**	Mangrove-channel	<1	26.76750 N	33.96283 E	611	0.35	0.42	0.90 ± 0.02
**95-31**	Reef slope	12	26.82933 N	33.98483 E	2301	0.25	0.59	0.86 ± 0.03
**B-5-8**	Sandy seagrass	6	26.82683 N	33.95383 E	3108	0.30	0.50	0.73 ± 0.05
**C-1-3**	Muddy sand with seagrass	40	26.83000 N	33.98683 E	4936	0.35	0.42	0.80 ± 0.06

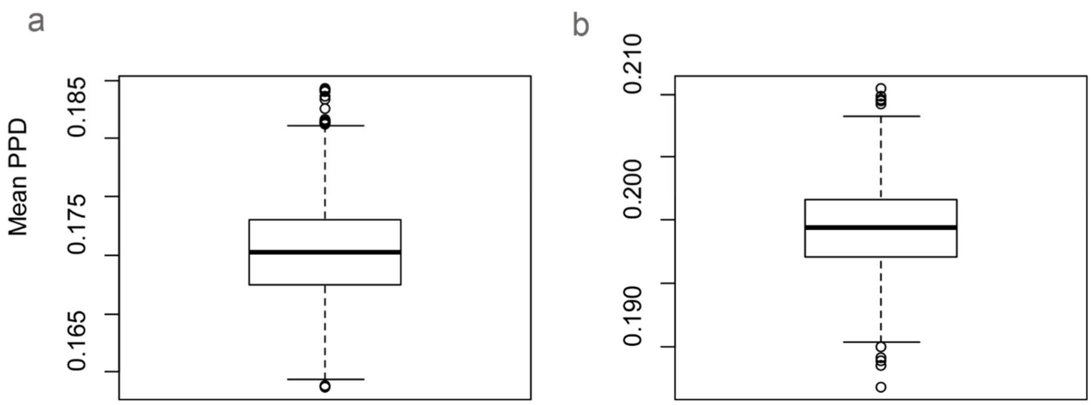

Fig. S4: Null model of mean beta diversity of the by-site (a) and the by-sample (b) Safaga dataset. Null model created by randomly sampling the gamma species pool until the number of specimens and number of sampling sites of the original datasets were obtained. Beta diversity was calculated as mean proportional dissimilarity over 1000 iterations.

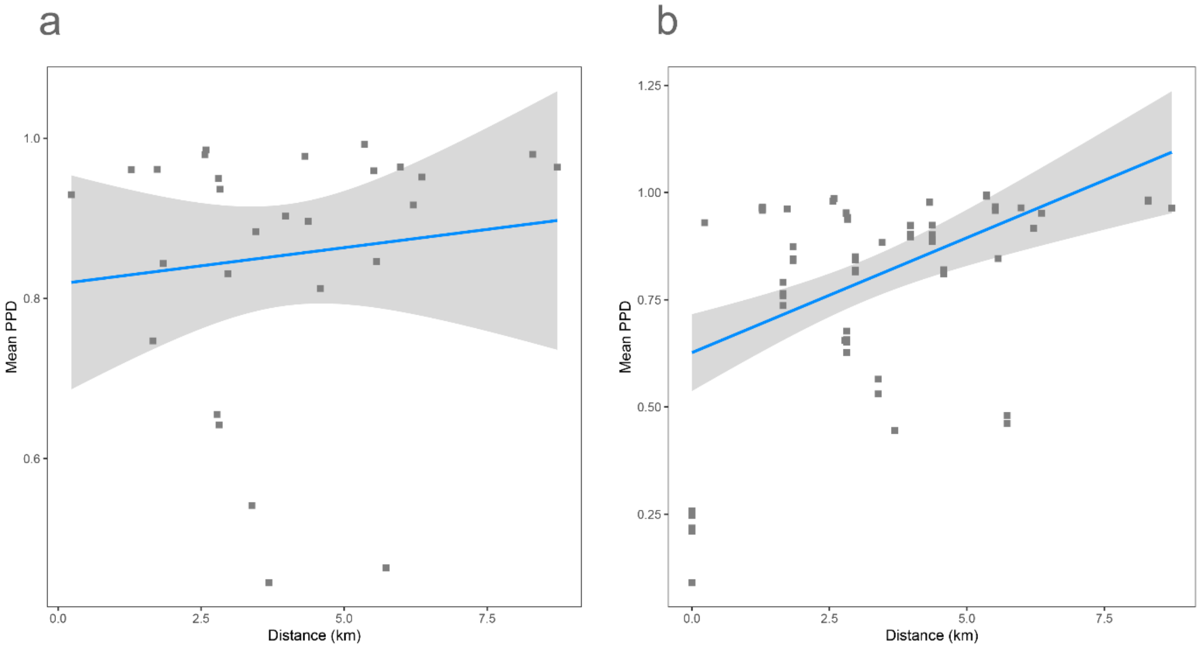

Fig. S5: Correlation between geographical distance and pairwise proportional dissimilarity in the by-site (a) and the by-sample (b) Safaga dataset. Distance decay is non-significant for the by-site dataset (Pearson correlation = 0.12, p = 0.56, Spearman's rho = 0.13, p = 0.49) and moderate for the by-sample dataset (Pearson correlation = 0.45, p < 0.001, rho = 0.28, p = 0.01).

Table S3: Pairwise proportional dissimilarity (PPD) of the studied samples from the Bay of Safaga. Samples taken from same site (only several meters apart) are combined (=by-site dataset); their composition is very similar (mean PPD samples 94-1-a to -d: 0.23 ± 0.01, PPD samples 94-3-a and -b: 0.22, PPD samples 94-4-a and -b: 0.09).

		**94-1**	**94-3**	**94-4**	**94-5**	**94-6**	**95-31**	**B-5-8**	**C-1-3**
		Sand between coral patches	Muddy sand	Mud	Reef slope	Mangrove channel	Reef slope	Sandy seagrass	Muddy sand with seagrass
**94-1**	Sand between coral patches		0.90	0.96	0.90	0.81	0.75	0.64	0.84
**94-3**	Muddy sand			0.83	0.98	0.94	0.96	0.54	0.46
**94-4**	Mud				0.99	0.98	0.99	0.96	0.95
**94-5**	Reef slope					0.96	0.66	0.96	0.98
**94-6**	Mangrove channel						0.92	0.85	0.95
**95-31**	Reef slope							0.88	0.93
**B-5-8**	Sandy seagrass								0.45
**C-1-3**	Muddy sand with seagrass								

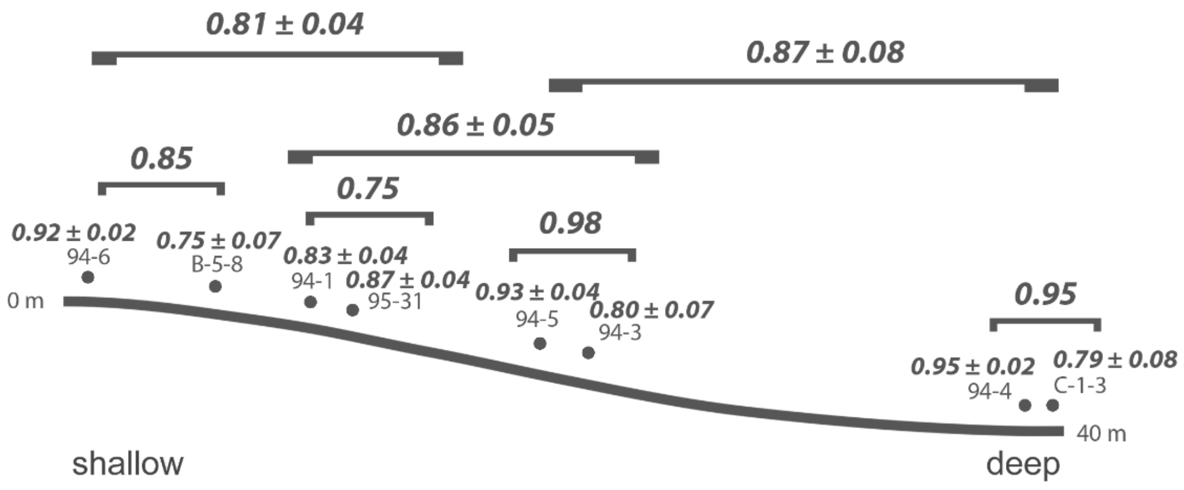

Fig. S6: Mean PPD among samples and depth categories for the by-site Safaga dataset.

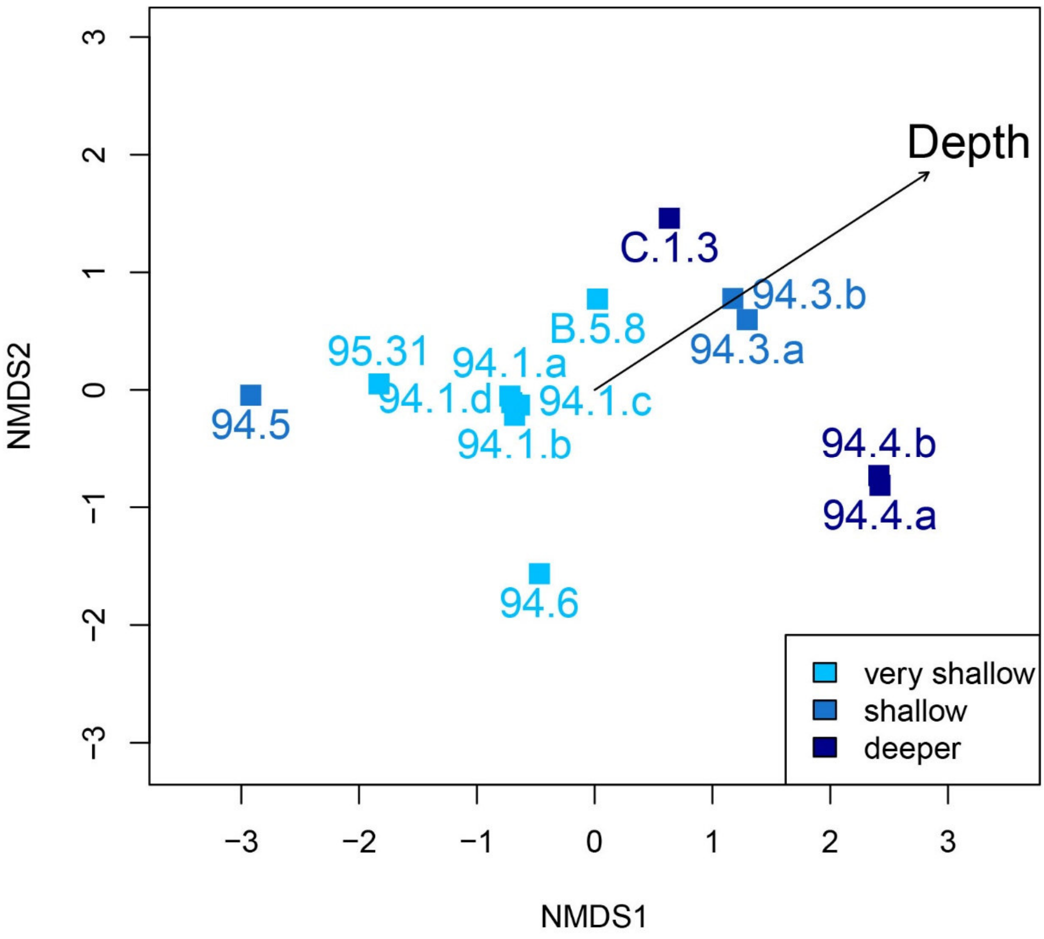

Fig. S7: Non-metric multidimensional scaling of the by-sample Safaga dataset, color-coded by depth. No strong relationships among localities from similar depths are seen. Arrow represents fitting of environmental factor depth as bathymetric gradient.

Part IV: Results from Safaga dataset, by sample

When samples from the same site are not combined (=by-sample dataset), overall beta diversity in the Safaga dataset is lower (0.82 ± 0.04; range: 0.05-1.00) than in the by-site dataset. A null model created from the gamma species pool of the by-sample dataset yields much lower beta diversity, with a mean of 0.12 ± 0.0001 (Fig. S8). Samples taken from same site (only several meters apart) are pooled in the by-site dataset (main text); their composition is very similar (mean PPD samples 94-1-a to -d: 0.12 ± 0.01, PPD samples 94-3-a and -b: 0.16, PPD samples 94-4-a and -b: 0.06). There is a low correlation (rho: 0.25, p-value: 0.02; Fig. S9) in the by-sample Safaga dataset, but this is attributed to very high similarities between samples from the same site.

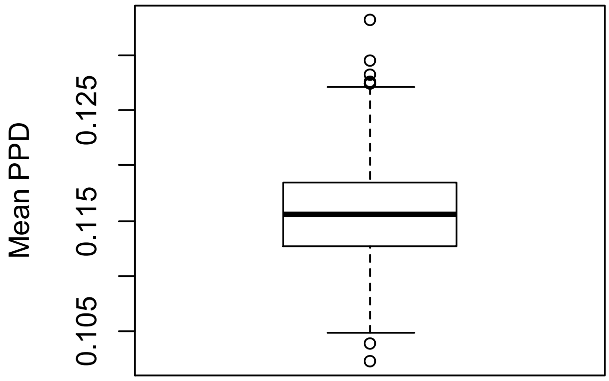

Fig. S8: Null model of mean beta diversity of the by-sample Safaga dataset. Beta diversity was calculated as mean proportional dissimilarity over 1000 iterations. The dataset was reduced to the ten most abundant species.

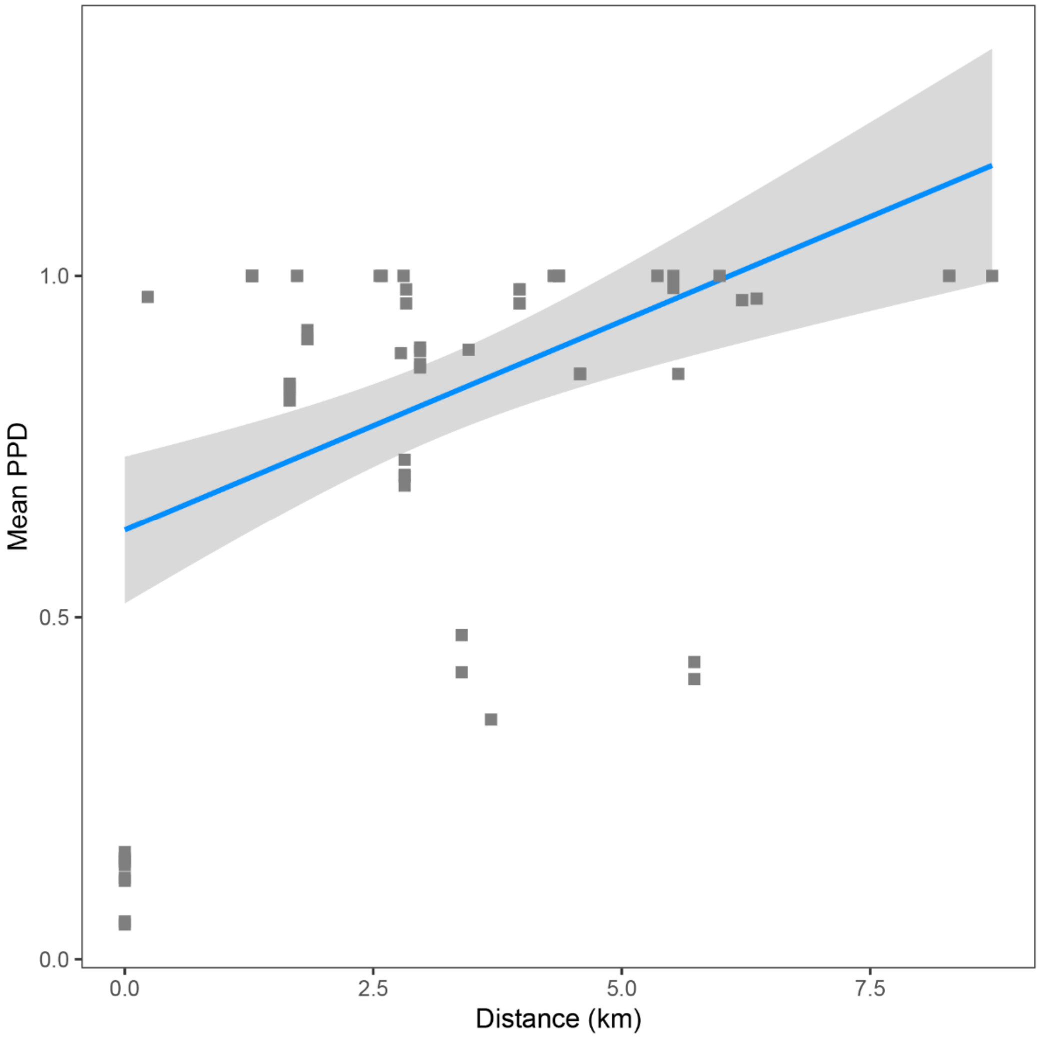

Fig. S9: Correlation between geographical distance and pairwise proportional dissimilarity in the by-sample Safaga dataset. Distance decay is low (Spearman's rank correlation rho: 0.25, p-value: 0.02). The dataset was reduced to the ten most abundant species.

Part V: Dispersion of homogeneity

Depicting homogeneity of dispersion using PPD, the Cassian data show relatively evenly dispersed sites (Fig. S10a), while the centroid using the modified Gower measure shows a slightly different dispersion (Fig. S10b). This is probably due to differences in dissimilarity metrics and methods of calculating the centroid, which is detailed in Anderson (2006). When samples are divided into two groups based on inferred water depth, there is no overlap of centroid or convex hull when applying PPD (Fig. S10c), although two samples (Lago Antorno and Misurina) plot very closely. This may be due to true ecological similarity despite differences in water depth, misinterpretation of water depth, or differences in taxonomic interpretation (both samples were identified by the same author). With the Gower measure, the two groups overlap (Fig. S10d), which corroborates our interpretation of water depth not being the main factor in driving differences in community composition.

Results are very similar for the Safaga dataset (Fig. S11). The sites are relatively evenly dispersed and the grouping according to (measured) water depth depends on the measure applied, with PPD yielding two separate groups and the Gower measure yielding overlapping centroids.

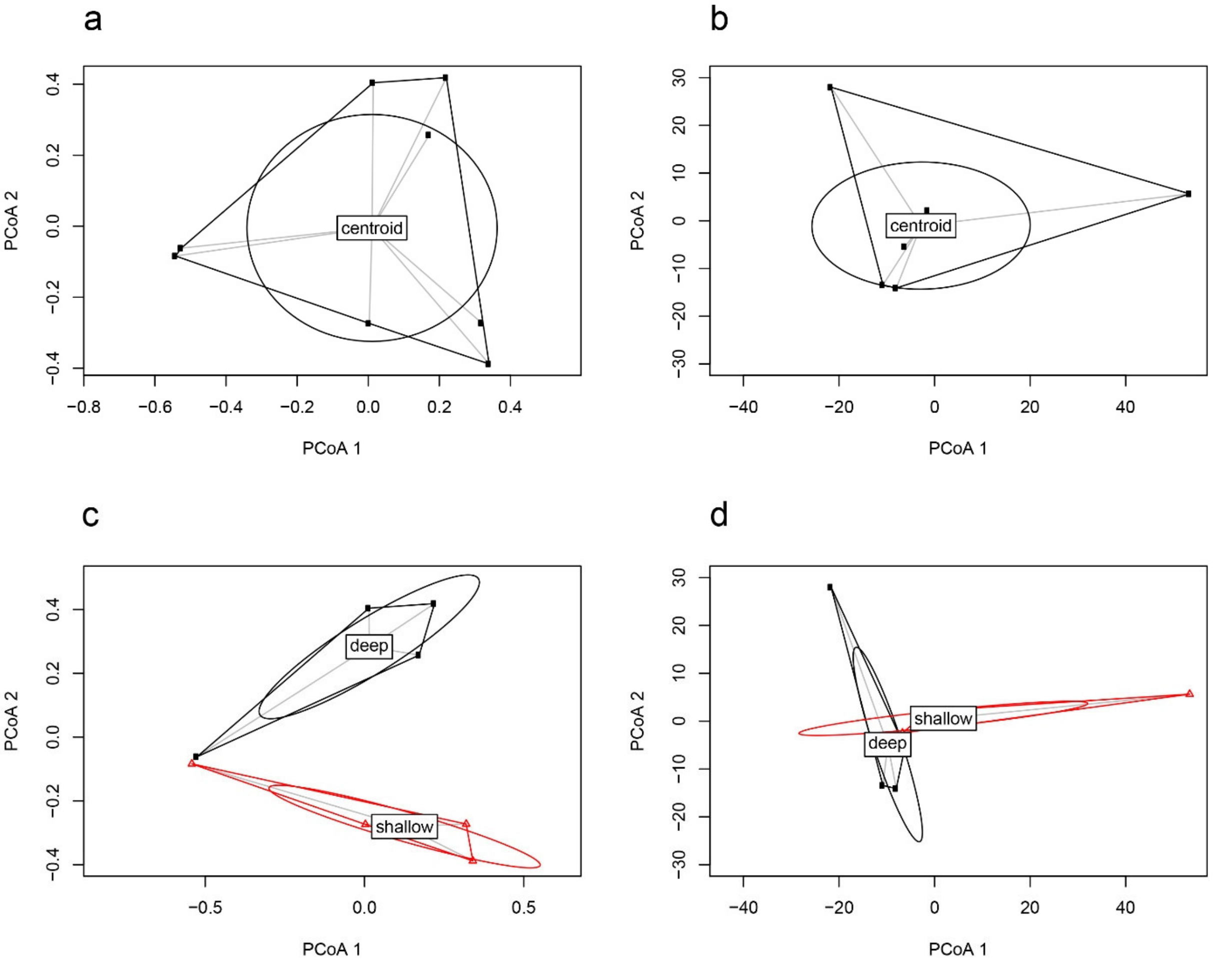

Fig. S10: Dispersion of homogeneity among the Cassian assemblages using pairwise proportional dissimilarity (a, c) and the modified Gower measure (b, d). Average distance to the group median is (a) 0.61, (b) 23.7, (c) deep: 0.54, shallow: 0.53, (d) deep: 21.7, shallow: 21.1.

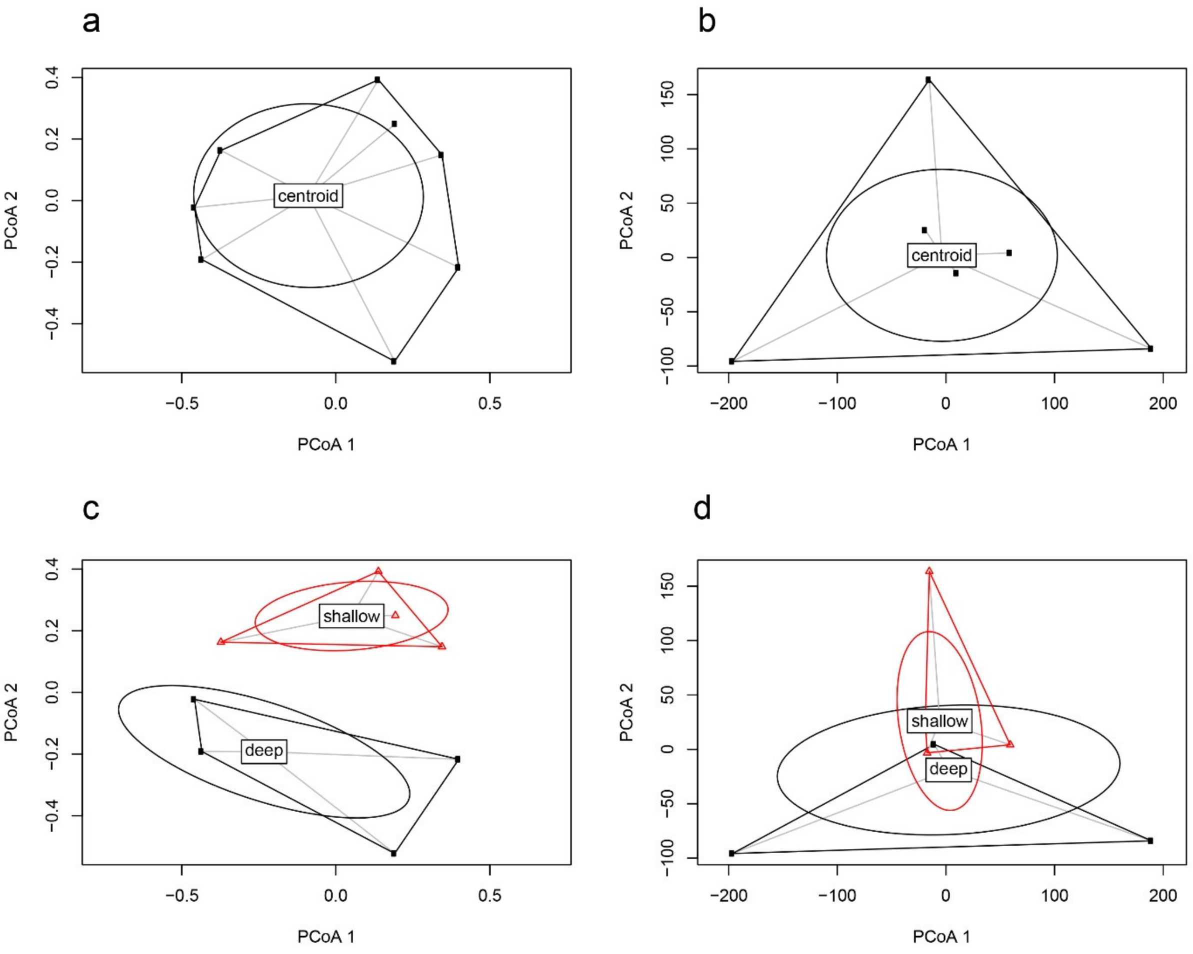

Fig. S11: Dispersion of homogeneity among the Safaga assemblages using pairwise proportional dissimilarity (a, c) and the modified Gower measure (b, d).

Part VI: Alpha diversity

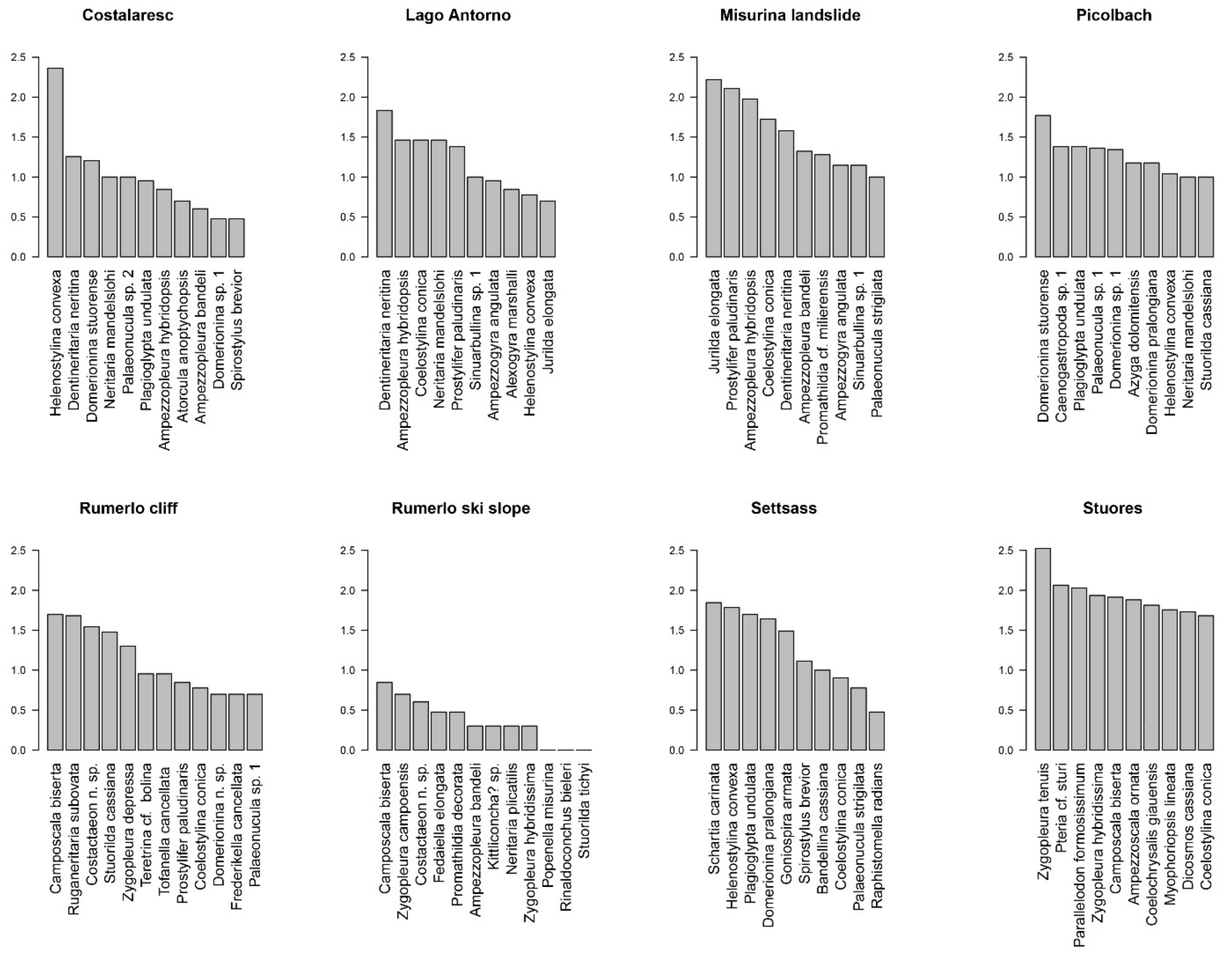

Fig. S12: Rank-abundance distributions of the 10 most abundant species in Cassian samples. Abundances logged (base 10).

Part VII: Distribution of pairwise values

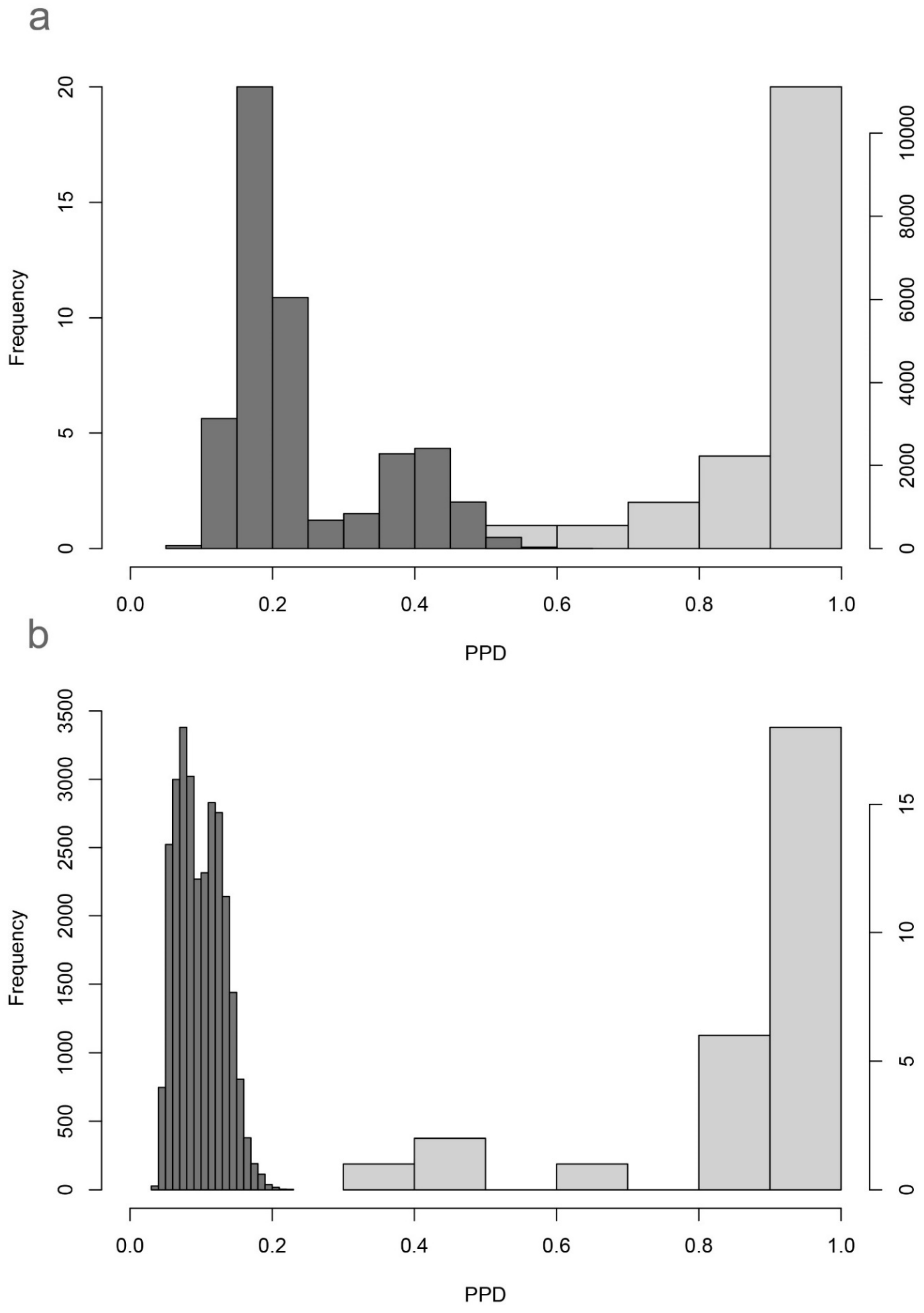

Fig. S13: Frequency distributions of pairwise proportional dissimilarity (PPD) values of (a) the Cassian dataset and (b) the by-site Safaga dataset. Light grey represents true values, dark grey represents values from respective null model.

Part VIII: Plates

Plates show the most abundant species from the Cassian sites Rumerlo ski slope, Costalaresc, Picolbach, and Rumerlo cliff (Figs. S14-16).

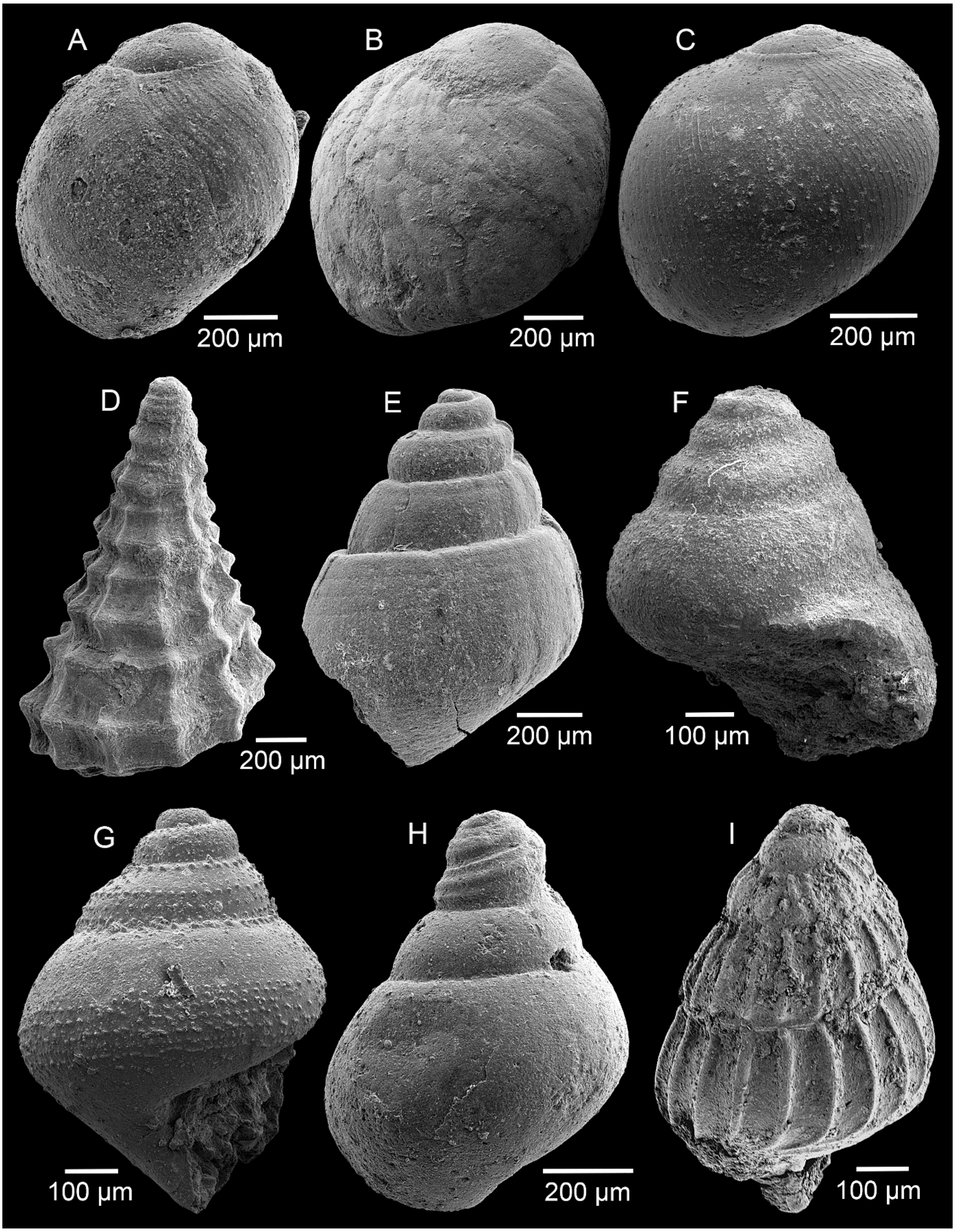

Fig. S14: (A) *Neritaria mandelslohi*, (B) *Ruganeritaria subovata*, (C) *Dentineritaria neritina*, (D) *Camposcala biserta*, (E) *Coelostylina conica*, (F) Caenogastropoda sp. 1, (G) *Helenostylina convexa*, larval shell, (H) *Prostylifer paludinaris*, (I) *Ampezzopleura hybridopsis*, larval shell.

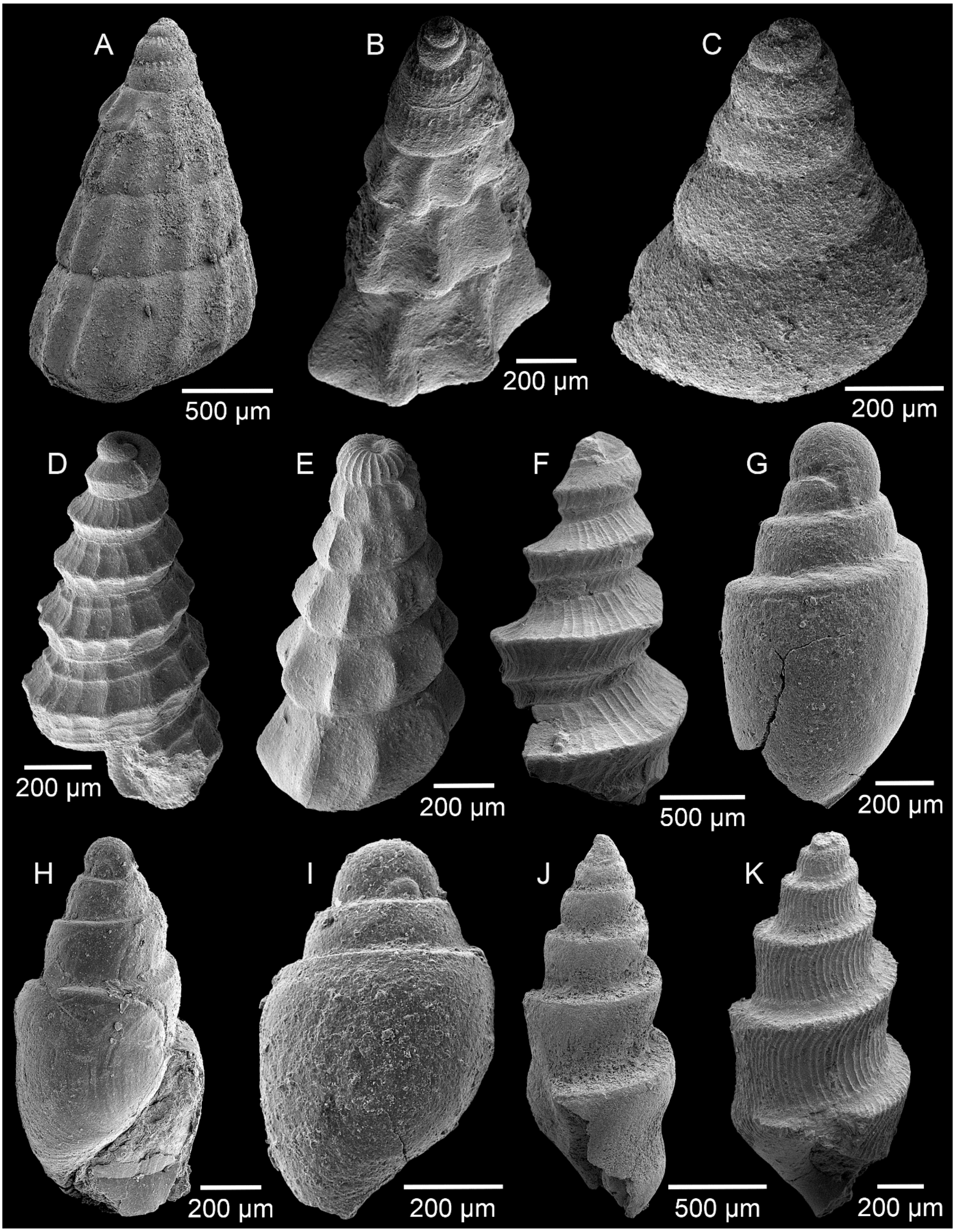

Fig. S15: (A) *Zygopleura campoensis*, (B) *Zygopleura depressa*, (C) *Azyga dolomitensis*, (D) *Tofanella cancellata*, (E) *Stuorilda cassiana*, (F) *Teretrina* cf. *bolina*, (G) *Domerionina pralongiana*, (H) *Domerionina stuorense*, (I) *Domerionina* sp. 1, (J) *Costactaeon* n. sp. (heavily encrusted), (K) *Costactaeon* n. sp.

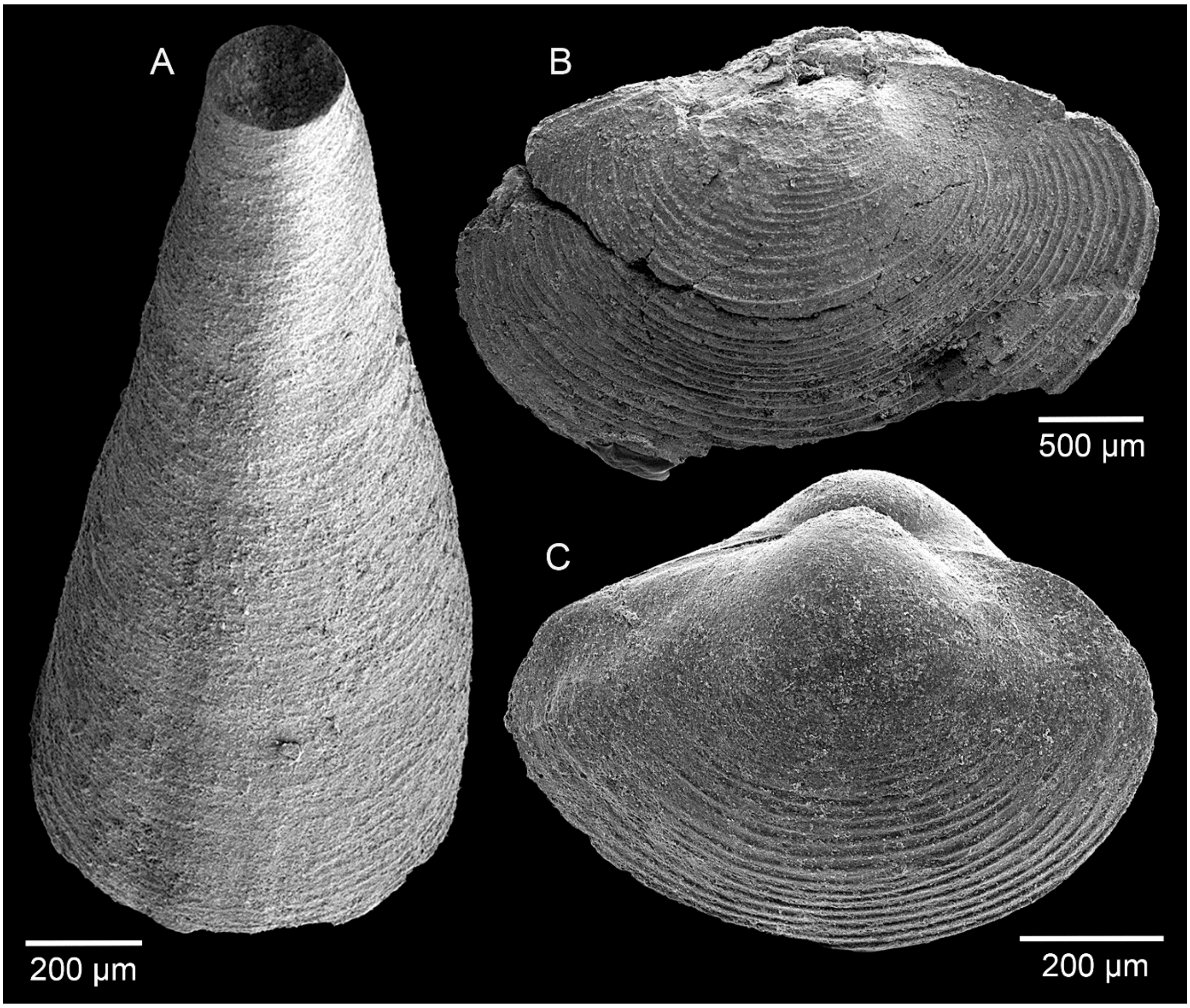

Fig. S16: (A) *Plagioglypta undulata*, (B) *Palaeonucula* sp. 1 (maybe juvenile *Prosoleptus lineatus*), (C) *Palaeonucula* sp. 2 (maybe juvenile *Prosoleptus lineatus*).

Most of the specimens from the analysed bulk samples are juveniles, fragments, or (more rarely) isolated protoconchs. Larger specimens with all ontogenetic stages preserved are rare. This represents a major handicap for the identification, especially because, by far, most of the previously described species are based on relatively large type specimens lacking the early ontogenetic shell including the protoconch. Therefore, linking juvenile specimens with the historic taxa is problematic in many cases. For instance, Zygopleuridae can, for the most part, only be identified if the protoconch is known. However, most of the species assigned to this family are based on type material that consists of teleoconch fragments. Many of such taxa will turn out to be nomina dubia. Other groups such as *Domerionina and Sinuarbullina* are seemingly diverse, but the differences between species are subtle and some of the species involved suffer from insufficient first descriptions and documentations. Thus, species identification is far from being trivial.

For the purpose of the paper, we made a great effort to at least be internally consistent in species identification. It seems to be crucial to illustrate at least the most abundant species so that our identifications become explicit and falsifiable. In the long run, all species must be characterized based on well preserved specimens representing all growth stages, including a re-study of the type material. However, this exceeds the scope of this study.

REFERENCES

Anderson MJ. 2006. Distance-based tests for homogeneity of multivariate dispersions. *Biometrics* 62:245-253.

Bizzarini F, and Laghi GF. 2005. La successione "Cassiana" nell'area a nord di Misurina (Trias, Dolomiti). *Lavori Società Veneziana Scienze Naturali* 30:127-143.

Fürsich FT, and Wendt J. 1977. Biostratinomy and palaeoecology of the Cassian Formation (Triassic) of the Southern Alps. *Palaeogeography, Palaeoclimatology, Palaeoecology* 22:257-323.

Gradstein FM, Ogg JG, Schmitz M, and Ogg G. 2012. *The Geologic Time Scale 2012*. Amsterdam: Elsevier.

Keim L, Spötl C, and Brandner R. 2006. The aftermath of the Carnian carbonate platform demise: a basinal perspective (Dolomites, Southern Alps). *Sedimentology* 53:361-386.

Nose M, Schlagintweit F, and Nützel A. 2018. New Record of Halimedacean Algaa from the Upper Triassic of the Southern Alps (Dolomites, Italy). *Rivista Italiana di Paleontologia e Stratigrafia (Research In Paleontology and Stratigraphy)* 124:421-431.

Roden VJ, Kocsis ÁT, Zuschin M, and Kiessling W. 2018. Reliable estimates of beta diversity with incomplete sampling. *Ecology* 99:1051-1062.

Roden VJ, Hausmann IM, Nützel A, Seuss B, Reich M, Urlichs M, Hagdorn H, and Kiessling W. 2019. Data from: Fossil liberation: a model to explain high biodiversity in the Triassic Cassian Formation, Dryad, Dataset, https://doi.org/10.5061/dryad.6s5651k.

Urlichs M. 2017. Revision of some stratigraphically relevant ammonoids from the Cassian Formation (latest Ladinian-Early Carnian, Triassic) of St. Cassian (Dolomites, Italy). *Neues Jahrbuch für Geologie und Paläontologie - Abhandlungen* 283:173-204.
